# Examination of shared gut microbiome signatures in aging and Parkinson’s disease

**DOI:** 10.3389/fnagi.2026.1745455

**Published:** 2026-03-13

**Authors:** Teddy Jia Wei Tng, Sarivin Vanan, Eng-King Tan, Li Zeng, Wilson Wen Bin Goh, Sunny Hei Wong, Kah-Leong Lim

**Affiliations:** 1 Lee Kong Chian School of Medicine, Nanyang Technological University, Singapore, Singapore; 2 Interdisciplinary Graduate Programme (IGP-Neuroscience), Nanyang Technological University, Singapore, Singapore; 3 National Neuroscience Institute, Singapore, Singapore; 4 Neuroscience and Behavioral Disorders Program, DUKE-NUS Graduate Medical School, Singapore, Singapore; 5 School of Biological Sciences, Nanyang Technological University, Singapore, Singapore; 6 Centre for Biomedical Informatics, Singapore, Singapore; 7 Centre for Microbiome Medicine, Nanyang Technological University, Singapore, Singapore

**Keywords:** aging, bacterial metabolites, butyrate, gut microbiome, Parkinson’s disease

## Abstract

Parkinson’s disease (PD) is a prevalent neurodegenerative disorder that is characterized clinically by a constellation of motoric deficits including resting tremors, bradykinesia, and rigidity. In recent years, there has been increasing interest in the gut-brain axis with several studies examining the relationship between gut microbiome and PD. Although association studies have reported multidimensional microbiome changes in PD, these observed changes may be confounded by various factors, especially age. Notably, existing literature on gut microbiome tends to consider aging and PD separately. This review thus examines the gut microbiome factors associated with both aging and PD. Our comprehensive analysis of the available literature reveals significant overlaps in gut microbes that are associated with aging and PD. For example, the bacterial genera *Akkermansia*, and *Alistipes* have shown increased abundance in both conditions, while *Faecalibacterium* and *Blautia* conversely show decreased abundance. Our findings were temporally consistent with more recent studies. These shared gut microbiome signatures were identified in patients across the clinical spectrum of PD symptom severity, and may influence aging and disease pathogenesis via depletion of butyrate, a beneficial anti-inflammatory microbial metabolite, since major producers of butyrate (such as *Faecalibacterium* and *Blautia*) were constantly decreased with age (across both Asian and Western populations). Given these observations, we wish to highlight the need to consider age-related factors in understanding microbiome changes in PD; the intersection of which could reveal gut microbes and their corresponding microbial metabolites such as butyrate as potential therapeutic targets for PD.

## Introduction

Parkinson’s disease (PD) is a common age-related neurodegenerative disorder whose prevalence is increasing rapidly in tandem with global population aging. The number of diagnosed PD patients has doubled over the past 3 decades and is expected to double again by year 2040 (Dorsey et al., 2023). Pathologically, PD is characterized by the loss of dopaminergic neurons (Langley et al., 2020) in the substantia nigra pars compacta (SNpc) of the midbrain that results in classical motor symptoms such as bradykinesia, rigidity, and resting tremors, which are behavioral markers for clinical diagnosis (Postuma et al., 2015; Moustafa et al., 2016). However, patients typically experience a prodromal period that is characterized by non-motor symptoms such as depression, constipation, REM sleep disorder, and olfactory loss (Haehner et al., 2011; Postuma et al., 2015; Liu et al., 2017) before the overt onset of movement deficits. The disease progression and symptom severity are clinically tracked using Hoehn and Yahr (H&Y) staging (Zhao et al., 2010) or the Unified Parkinson’s Disease Rating Scale (UPDRS) including the Movement Disorder Society (MDS) UPDRS (Movement Disorder Society Task Force on Rating Scales for Parkinson’s Disease, 2003; Goetz et al., 2008), with increasing score indicating worsening PD disability for both assessments (Holden et al., 2018).

The histopathological hallmark of PD is the accumulation of α-synuclein, a neuronal protein, into aggregates called Lewy bodies in the SNpc (Mackenzie, 2001). In 2003, the German pathologist Heiko Braak postulated that sporadic PD could develop from the spreading of these α-synuclein aggregates starting either from the nasal cavity or the gut before they infiltrate the brain (Rietdijk et al., 2017; Borghammer and Van Den Berge, 2019; Van Den Berge and Ulusoy, 2022). Supporting Braak’s hypothesis, there is now accumulating evidence suggesting an important role of the gut-brain axis in PD risk and progression (Tan et al., 2022). Indeed, studies have demonstrated the spreading of α-synuclein pathology from the gut to the brain via the vagus nerve connecting the two organs (Kim S. et al., 2019). Additionally, gut microbiota from PD patients worsens physical impairments when colonized into a PD mouse model (Sampson et al., 2016). Thus, the gut microbiome could play a role in PD pathogenesis.

Generally, PD risk factors include genetic predisposition (e.g., *Pink1, Parkin, LRRK2* mutations) (Billingsley et al., 2018), head trauma (Bower et al., 2003) and exposure to environmental neurotoxicants such as pesticides (Kamel et al., 2007). However, exposure to such environmental factors are rather selective to certain populations, and genetics only account for 3–5% of all PD patients (Alafifi et al., 2020). The remaining 95% of PD cases are sporadic in nature, where aging remains the biggest contributing risk factor (Collier et al., 2011). Loss of neurons is common with aging; however, dopaminergic neurons in the SNpc are observed to be preferentially vulnerable to degeneration at a rate much higher than other neurons in the brain (Reeve et al., 2014). One study with 750 elderly (non-PD) participants showed that at least one-third of the study population displayed mild to severe neuronal loss in the SNpc, and 10% exhibited characteristic Lewy body pathology post-mortem (Buchman et al., 2012). Such vulnerability can be attributed to the accumulation of oxidative stress, DNA damage, dysfunctional mitochondria and protein aggregates that are a result of deteriorating cellular maintenance that comes with aging (Reeve et al., 2014). It also emphasizes the intimate relationship between aging and PD.

Studied have identified the gut microbiome as one factor that could be associated with the onset and progression of PD. The known relationship between the gut microbiome and PD is complex; studies suggesting that this relationship is casual (i.e., gut microbiome accelerates PD development) and studies proposing that the relationship is consequential both exist in current literature. As alluded to earlier, Sampson et al. (2016) published a seminal study highlighting the evidence for a causal link; here gut microbiota collected from PD patients sufficiently aggravated motor deficits when colonized into a PD mouse model (Sampson et al., 2016). Additional studies support this causal relationship (Ning et al., 2022; Zhu et al., 2022; Chen et al., 2025). On the other hand, different studies show that altered gut microbiome is a consequence of PD; medications for PD such as L-Dopa (Maini Rekdal et al., 2019; Zhong et al., 2023) and prodromal PD symptoms such as constipation (Yang et al., 2022) have been shown to change the gut microbiome. Various studies advocating for the consequential relationship show that manipulating the gut microbiome has minimal downstream effect on PD development (Scheperjans et al., 2024; De Sciscio et al., 2025). It should be noted that despite the exact nature of the relationship between gut microbiome and PD (casual or consequential) remaining uncertain, the association between the gut microbiome and PD has been shown to be robust. Recognizing this, metagenomic sequencing studies related to PD gut microbiome have been conducted across various ethnic groups and/or countries (Table 1). Although many of these studies have addressed confounding factors, such as dietary pattern, geography, medicine-use or comorbidities, the confounding effect of age is of particular relevance given its a major risk factor for PD and as well as a key determinant of gut microbiome composition (Hindle, 2010). Given this, we seek to elucidate a working model of the gut microbiome’s relationship that considers both aging and PD. Additionally, since dietary variations across different ethnic groups can affect the composition of gut microbiome (Leeming et al., 2019), we also examined the presence of unique subsets of PD gut microbiota that are linked with ethnicity.

**TABLE 1:** Compilation of PD papers analyzed (2017–2022).

No.	Paper	Authorship year	Sample	Type	Control No.	PDNo.	Race	Age range	PD Staging (score if available)	Data availability
Pl	Implications of the Gut Microbiome in Parkinson’s Disease	Elfil et al., 2020	NIL	Review	NIL	NIL	NIL	NIL	NIL	NIL
P2	Altered gut microbiota and inflammatory cytokine responses in patients with Parkinson’s disease	Lin et al., 2019	Fecal	Article	77	80	Taiwan	62–64	HY(1.8–2.6)	Upon request
P3	Meta-Analysis of Gut Dysbiosis in Parkinson’s Disease	Nishiwaki et al., 2020	Fecal	Meta-analysis	137	223	Japan	NIL	NIL	NIL
P4	Gut microbiome in Parkinson’s disease: New insights from meta-analysis	Toh et al., 2022	Fecal	Meta-analysis	734	969	Caucasian/Non-Caucasian	62–70	UPDRS lll-IV scoring	Majority available in the NCBI Gen Bank
PS	Parkinson’s disease and Parkinson’s disease medications have distinct signatures of the gut microbiome	Hill-Burns et al., 2017	Fecal	Article	130	197	US	NIL	UPDRS III scoring	ERP016332
P6	The gut microbiome in Parkinson’s disease [In German]	Bedarf et al., 2019	Unknown	Article	Unknown	Unknown	Germany	NIL	NIL	NIL
P7	Gut microbiota in Parkinson disease in a northern German cohort	Hopfner et al., 2017	Fecal	Article	29	29	Germany	69 ± 7	UPDRS III scoring (21)	NIL
P8	Alteration of the fecal microbiota in Chinese patients with Parkinson’s disease	Qian et al., 2018	Fecal	Article	45	45	China	68 ± 8	UPDRS III scoring (22)	PRJNA391524
P9	Gut microbiota in patients with Parkinson’s disease in southern China	Lin et al., 2018	Fecal	Article	45	75	China	60 ± 10	UPDRS III scoring (34)	NIL
PIO	Gut Microbiota Differs Between Parkinson’s Disease Patients and Healthy Controls in Northeast China	Li C. et al., 2019	Fecal	Article	48	51	China	62 ± 9	UPDRS III scoring (24)	NIL
PII	The nasal and gut microbiome in Parkinson’s disease and idiopathic rapid eye movement sleep behavior disorder	Heintz-Buschart et al., 2018	Fecal	Article	78	76	Germany	68 ± 10	UPDRS III scoring (30)	PRJNA381395
P12	Gut Microbial Ecosystem in Parkinson Disease: New Clinicobiological Insights from Multi-Omics	Tan et al., 2021	Fecal	Article	96	104	Malaysia/Asian	65 ± 9	UPDRS III scoring (31)	PRJNA494620
P13	Unraveling gut microbiota in Parkinson’s disease and atypical parkinsonism	Barichella et al., 2019	Fecal	Article	113	193	Italian	66 ± 10	UPDRS III scoring (17)	NIL
P14	Gut microbiota in Parkinson’s disease: Temporal stability and relations to disease progression	Aho et al., 2019	Fecal	Article	64	64	Finland	65 ± 6	UPDRS	PRJEB27564
P15	Dysbiosis of gut microbiota in a selected population of Parkinson’s patients	Pietrucci et al., 2019	Fecal	Article	72	80	Italy	66 ± 9	UPDRS	PRJNA510730
P16	Microbiota Composition and Metabolism Are Associated With Gut Function in Parkinson’s Disease	Cirstea et al., 2020	Fecal	Article	103	197	Canada	66 ± 5	UPDRS III scoring (21)	NIL
P17	Characterizing dysbiosis of gut microbiome in PD: evidence for overabundance of opportunistic pathogens	Wallen et al., 2020	Fecal	Meta-analysis	320	535	US	NIL	NIL	PRJNA601994
P18	Meta-analysis of the Parkinson’s disease gut microbiome suggests alterations linked to intestinal inflammation	Romano et al., 2021	Fecal	Meta-analysis	NIL	NIL	Across 6 countries	60–70	UPDRS III	Majority publicly available
P19	Nutritional Intake and Gut Microbiome Composition Predict Parkinson’s Disease	Lubomski et al., 2022a	Fecal	Article	81	103	Sydney	67 ± 12	UPDRS III scoring (32.9)	PRJNA808166
P20	The Association Between the Gut Microbiota and Parkinson’s Disease, a Meta-Analysis	Shen et al., 2021	Fecal	Meta-analysis	NIL	NIL	Across 6 countries	60–76	NIL	NIL
P21	Functional implications of microbial and viral gut metagenome changes in early stage L-DOPA-naïve Parkinson’s disease patients	Bedarf et al., 2017	Fecal	Article	28	31	Germany	65 ± 10	UPDRS III scoring (12.6)	ERP019674
P22	Analysis of Gut Microbiota in Patients with Parkinson’s Disease	Petrov et al., 2017	Fecal	Article	No full text access					
P23	Structural changes of gut microbiota in Parkinson’s disease and its correlation with clinical features	Li et al., 2017	Fecal	Article	14	24	China	74 ± 6	NIL	NIL
P24	Alteration of the fecal microbiota in North-Eastern Han Chinese population with sporadic Parkinson’s disease	Li F. et al., 2019	Fecal	Article	10	10	China	80 ± 8	Total UPDRS scoring (42)	NIL
P25	Gut Microbiota Altered in Mild Cognitive Impairment Compared With Normal Cognition in Sporadic Parkinson’s Disease	Ren et al., 2020	Fecal	Article	13	14	China	60 ± 9	UPDRS III (30)	PRJNA561023
P26	Parkinson’s disease-associated alterations of the gut microbiome predict disease-relevant changes in metabolic functions	Baldini et al., 2020	Fecal	Article	162	147	Luxembourg	69 ± 8	UPDRS III (35)	Upon request
P27	Effect of Parkinson’s disease and related medications on the composition of the fecal bacterial microbiota	Weis et al., 2019	Fecal	Article	25	34	Germany	68 ± 9	H&Y staging	PRJEB30615
P28	Analysis of the Gut Microflora in Patients With Parkinson’s Disease	Jin et al., 2019	Fecal	Article	68	72	China	65 ± 4	UPDRS	Accession no.: 13258423–13258555
P29	Altered gut microbiota in Parkinson’s disease patients/healthy spouses and its association with clinical features	Zhang et al., 2020	Fecal	Article	74	63	China	Majority 52–74	H&Y staging	CRA001938
API	Gut Microbiota Dysbiosis Is Associated with Elevated Bile Acids in Parkinson’s Disease	Li P. et al., 2021	Appendix	Article	12	15	Oregon US brain bank	53–92	Braak(5–6)	GSE135743
P30	Parkinson’s Disease and the Gut Microbiome in Rural California	Zhang K. et al., 2022	Fecal	Article	74	96	US	No full text access		
P31	Oral, Nasal, and Gut Microbiota in Parkinson’s Disease	Li et al., 2022	Fecal	Article	75	78	China	65 ± 6	UPDRS III (28)	NIL
P32	Urolithins: potential biomarkers of gut dysbiosis and disease stage in Parkinson’s patients	Romo-Vaquero et al., 2022	Fecal	Article	117	52	Spain	68 ± 8	H&Y staging	NIL
P33	Oral and gut dysbiosis leads to functional alterations in Parkinson’s disease	Jo et al., 2022	Fecal	Article	85	91	Korea	65 ± 8	UPDRS III (32)	PRJNA742875 and PRJNA743718
P34	Fecal microbiome alterations in treatment-naive de novo Parkinson’s disease	Boertien et al., 2022	Fecal	Article	85	136	Europe	65 ± 10	UPDRS III (32)	PRJEB55464
										Mild PD
										Moderate PD

## Methods

We examined studies that employed 16S rRNA sequencing given the larger number of microbiome studies in PD and aging (Janda and Abbott, 2007). To get insights into recent developments in the field, papers on 16S rRNA sequencing of fecal samples from PD patients were gathered via PubMed in a publishing window of 2017–2022. The search term “parkinson’s + gut microbiome” was used. This yielded 689 results. Only original articles and meta-analyses that reported differentially abundant gut microbiome in human samples were selected. Review papers were examined for the original research articles that were cited. A total of 35 papers were examined for this review (Table 1; Bedarf et al., 2017; Hill-Burns et al., 2017; Hopfner et al., 2017; Li et al., 2017; Petrov et al., 2017; Heintz-Buschart et al., 2018; Lin et al., 2018; Qian et al., 2018; Aho et al., 2019; Barichella et al., 2019; Bedarf et al., 2019; Jin et al., 2019; Li C. et al., 2019; Li F. et al., 2019; Lin et al., 2019; Pietrucci et al., 2019; Weis et al., 2019; Baldini et al., 2020; Cirstea et al., 2020; Elfil et al., 2020; Nishiwaki et al., 2020; Ren et al., 2020; Wallen et al., 2020; Zhang et al., 2020; Li P. et al., 2021; Romano et al., 2021; Shen et al., 2021; Tan et al., 2021; Boertien et al., 2022; Jo et al., 2022; Li et al., 2022; Lubomski et al., 2022a; Romo-Vaquero et al., 2022; Toh et al., 2022; Zhang K. et al., 2022). It is interesting to note that the number of papers rose rapidly from just 48 papers in 2017 to over 200 papers per year in the last 3 years; this reinforces how PD research focus has increasingly embraced the gut-brain axis. A similar search process was done for aging-related studies using the search term “aging + gut microbiome” or “centenarian + gut microbiome.” Only original articles that studied gut microbiome differences between healthy young and healthy centenarians were selected, and those confounded with other experimental variables such as medication or supplement treatments were omitted. A total of 11 aging-related papers were examined for this review (Table 2; Maffei et al., 2017; Kim B. et al., 2019; Tuikhar et al., 2019; Wang et al., 2019; Wu et al., 2019; Badal et al., 2020; Palmas et al., 2022; Sepp et al., 2022; Wang J. et al., 2022; Wu J. et al., 2022; Wu L. et al., 2022). Additionally, we reviewed literature from the past 5 years (2021–2025) on the key PD-associated metabolite butyrate. A search using the terms “Parkinson’s” and “butyrate” yielded 67 publications, suggesting increasing interest in this metabolite.

**TABLE 2:** Compilation of aging-related papers analyzed (2017–2022).

No.	Paper	Authorship year	Sample	Type	Control (Young) No.	Aged no.	Centenarian no.	Race	Age range	Data availability
AG1	The Gut Microbiome, Aging, and Longevity: A Systematic Review	Badal et al., 2020	Intervention, Cognition, Centenarian Studies	Systematic Review	NIL					
AG2	Comparison of the gut microbiota of centenarians in longevity villages of South Korea with those of other age groups	Kim B. et al., 2019	Fecal	Article	9	17	30	Korean	26–43; 67–69; 95–108	PRJEB7507
AG3	Comparative analysis of the gut microbiota in centenarians and young adults shows a common signature across genotypically non-related populations	Tuikhar et al., 2019	Fecal	Article	30	0	30	India	28–47; 97–110	MG-RAST (http://metagenomics.anl.gov/linkin.cgi?project=16687).
AG4	A Cross-Sectional Study of Compositional and Functional Profiles of Gut Microbiota in Sardinian Centenarians	Wu et al., 2019	Fecal	Article	17	23	19	Sardinia	21–33; 68–88; 99–107	PRJEB25514
AG5	Enriched taxa were found among the gut microbiota of centenarians in East China	Wang et al., 2019	Fecal	Article	0	95	92	China	66–69; 92–99; > 100	NIL
AG6	Biological Aging and the Human Gut Microbiota	Maffei et al., 2017	Fecal	Article	Total 85		0	US	43–79	NIL
AG7	Gut microbiota as an antioxidant system in centenarians associated with high antioxidant activities of gut-resident Lactobacillus	Wu L. et al., 2022	Fecal	Article	52	158	18	China	80–120 vs. 20–60	PRJNA895352
AG8	The landscape in the gut microbiome of long-lived families reveals new insights on longevity and aging - relevant neural and immune function	Wang J. et al., 2022	Fecal	Article	11	0	32	China	16–52; 100–108	CNP0002519
AG9	Comparative Analysis of Gut Microbiota in Centenarians and Young People: Impact of Eating Habits and Childhood Living Environment	Sepp et al., 2022	Fecal	Article	25	0	25	Estonia	19–23; 96–105	PRJNA806961
AG10	Gut Microbiota Markers and Dietary Habits Associated with Extreme Longevity in Healthy Sardinian Centenarians	Palmas et al., 2022	Fecal	Article	46	29	17	Italy	42–58; 91–95; 100–104	PRJEB52843
AG11	Age-Related Changes in the Composition of Intestinal Microbiota in Elderly Chinese Individuals	Wu J. et al., 2022	Fecal	Article	37	83	36	China	35–79; 80–94; 95–102	NIL

Every paper recorded is given a unique key, P_x (where x is a numeric), for PD in Table 1, or AG_x, for aging in Table 2. These unique keys serve as headers for each list of microbiomes reported in Supplementary Tables 1, 2 for mapping of the data to the specific papers. The microbes’ identities were collated exactly as they were reported in the respective papers. The microbiomes reported were sorted and recorded according to whether there was an increase (Supplementary Table 1) or decrease (Supplementary Table 2) in abundance for each of these papers. The intersect (overlapping microbiome trends between PD and aging) and complement (microbiome trends unique to PD or aging) were then examined. Differences in gut microbiome across ethnicity (based on Race column in Table 1) and PD symptom severity (green for mild PD, pink for moderate PD in Table 1) were also studied. PD symptom severity was classified as mild for UPDRS III scores lower or equal to 32, moderate for scores between 33 and 58 and severe for scores above or equal to 59 (Martínez-Martín et al., 2015). Amongst the papers that have information on UPDRS III scores, 13 were mild and 4 were moderate. In addition, a Jaccard Index, represented as J(A,B) = | A ∩ B| /| A ∪ B|, was calculated to represent the similarity between PD and aging datasets. The Jaccard Distance (1-Jaccard Index) was also calculated to represent the dissimilarity between ethnicity datasets. All analyses were done using R (v1.4.1106).

## Summary of description of studies used

A total of 35 papers on PD gut microbiome and 11 papers on aging-related gut microbiome from the 5-year period between 2017 and 2022 were examined. Of the 35 PD papers, one analyzed appendix samples (Li P. et al., 2021) while the rest of the 34 used fecal samples. The 35 PD papers consist of one review, five meta-analyses, and 29 research articles; they cover patients from ages 52–88, with sample sizes from 10 up to 200 subjects, spanning across 12 different countries that capture both Asian and Western populations. Among the papers that provided a numerical score specifically for UPDRS III PD symptom severity grading, 13 were mild and 3 were moderate.

Similarly, the 11 aging-related papers analyzed fecal samples. These papers consist of one review and 10 articles covering young, elderly, and centenarian groups, with sample sizes of 9 subjects up to 158 subjects, spanning across 7 countries that capture both Asian and Western populations. All the aging studies only examined elderly and centenarians who are healthy and free from medical interventions to prevent confounding effects of other diseases and drugs. Many studies also attempt to compare the young and old from similar geographical proximity (e.g., from the same village) or from the same household to reduce confounding effects from dietary differences.

### Major overlaps of aging with PD-related gut microbiome reinforces aging as a major risk factor

Reported gut microbiomes from PD and aging studies were characterized based on increased or decreased abundance (Supplementary Tables 1, 2 respectively). The top 10 most commonly reported gut microbiome changes for both PD and aging are collated in Table 3 (*↑: increased abundance in both PD and aging, *↓: decreased abundance in both PD and aging). A comparison of the list of top hits from the 2 separate study populations revealed that *Akkermansia, Alistipes, Parabacteroides*, and *Butyricimonas* are frequently increased in abundance, while *Faecalibacterium, Lachnospiraceae*, and *Blautia* are usually decreased for both PD and healthy elderly populations. The Jaccard Index for top 10 increased microbes between PD and aging populations is 0.43, while that for decreased microbes is 0.19, suggesting that increased gut microbiome populations related to aging might be a risk factor for PD.

**TABLE 3:** Top 10 gut microbiome changes in PD and aging (2017–2022).

Microbiome	%Up PD	Microbiome	%Down PD	Microbiome	%Up Aging	Microbiome	%Down Aging
Akkermansia *↑	51.429	Roseburia	38.710	Akkermansia *↑	54.545	Faecalibacterium *↓	60.000
Bifidobacterium	31.429	Faecalibacterium *↓	35.484	Alistipes *↑	36.364	Bacteroides	40.000
Lactobacillus	28.571	Lachnospiraceae *↓	29.032	Methanobrevibacter	36.364	Blautia *↓	30.000
Alistipes *↑	22.857	Prevotella	22.581	Parabacteroides *↑	36.364	Eubacterium	30.000
Parabacteroides *↑	22.857	Blautia *↓	16.129	Butyricimonas *↑	27.273	Lachnospiraceae *↓	30.000
Butyricimonas *↑	20.000	Ruminococcus	16.129	Desulfovibrio *↑	27.273	Anaerostipes	20.000
Ruminococcaceae *↑	17.143	Fusicatenibacter	12.903	Eggerthella	27.273	Bacteroidaceae	20.000
Christensenella	14.286	Prevotellaceae	12.903	Odoribacter	27.273	Butyricicoccus	20.000
Desulfovibrio *↑	14.286	Unclassified Lachnospiraceae	12.903	Porphyromonas	27.273	Coprococcus	20.000
Megasphaera	14.286	Agathobacter	9.677	Ruminococcaceae *↑	27.273	Dorea	20.000

*↑ Up in both PD and aging. *↓ Down in both PD and aging.

**FIGURE 1:**
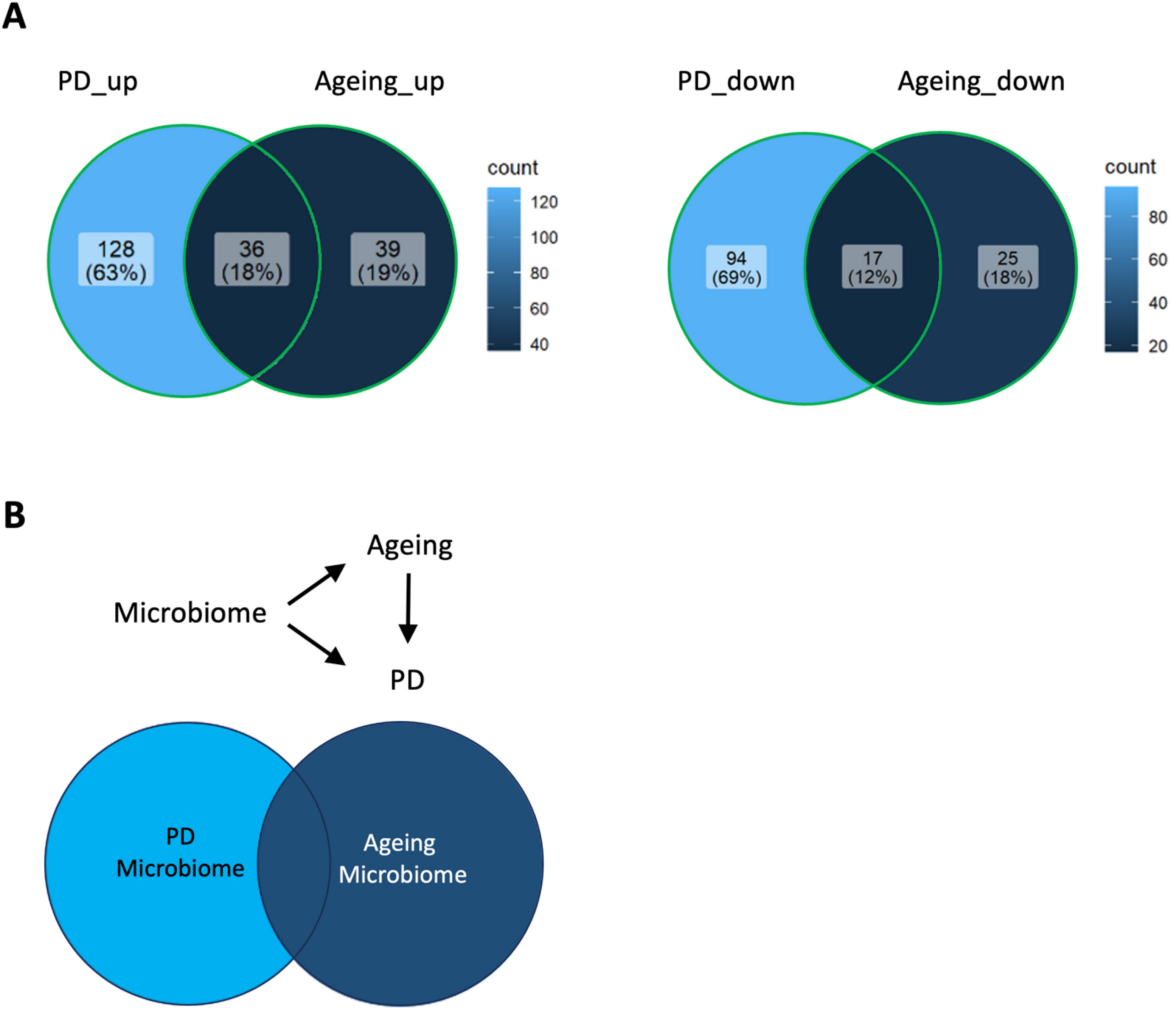
Gut microbiome relationship between aging and Parkinson’s disease. **(A)** Intersection analysis between aging and PD studies split by increased (left) and decreased (right) abundance. High percentage of aging related microbes overlapped with PD related microbes suggesting aging as a risk factor for PD. **(B)** Proposed model on gut microbiome relationship with aging and PD. Unique gut microbes that affect aging and PD independently and some confounded gut microbes that first affect aging then subsequently affect PD as indicated by the unidirectional arrow from aging to PD.

Next, the list of all gut microbiomes was pooled together for an intersection analysis. Of the gut microbes that were reported at least once in both PD and aging studies, the most frequently increased in PD and aging are *Akkermansia, Bifidobacterium, Lactobacillus, Alistipes*, and *Parabacteroides*. The top frequency hits for gut microbiome that increased in the PD unique complement are *Christensenella* and *Megasphaera*, while those for the aging unique complement are *Clostridium* and *Eggerthella*. On the other hand, of the gut microbes that were reported at least once in both PD and aging studies, the most frequently decreased in PD are *Roseburia, Faecalibacterium, Lachnospiraceae, Prevotella* and *Blautia*. The top frequency hits for gut microbiome that decreased in the PD unique complement are *Prevotellaceae* and *Agathobacter*. Interestingly, the intersection analysis of the aging and PD sets of gut microbiomes revealed that many microbiomes in the aging set (48% for increased abundance pool and 40% for decreased abundance pool) also contribute to PD, but the converse was not observed (Figures 1A,B).

We next pondered whether specific microbes are associated with severity of PD symptoms. Hence, we compared the microbiome for mildly symptomatic PD (UPDRS III below 33) and moderately symptomatic PD (UPDRS III between 33 and 58) noting that none of the studies examined reported severely symptomatic PD (UPDRS III above 58) (Martínez-Martín et al., 2015). Subsequently, intersection analysis was done to identify the different sets of gut microbiomes that correspond to symptom severity based on mild PD and moderate PD. Importantly, gut microbes that are associated with aging and PD did not appear to correlate with PD symptom severity (i.e., the microbes that overlap between PD and aging demonstrate similar changes for both mild and moderately symptomatic PD patients). Therefore there is no unique microbiomic observation that can classify PD patients of different clinical severity (Table 4).

**TABLE 4:** PD symptom severity and ethnicity’s relationship with bacterial taxa and associated metabolites.

Identity	PD/Aging	PD severity (direction, 35↑/31↓)	Ethnicity	Metabolite	Still reported in 2023–2025 window?
Akkermansia	PD and Aging	Mild and moderate (20↑)	Common	SCFA (acetic and butyric acid) and BCFA	Yes
Alistipes	PD and Aging	Mild and moderate (8↑)	More Asian	Sulfonolipid	Yes
Parabacteroides	PD and Aging	Mild and moderate (8↑)	More Asian	SCFA (acetate)	Yes
Odoribacter	PD and Aging	Mild and moderate (4↑)	Asian	SCFA (acetate, propionate, butyrate) and Sulfonolipid	Yes
Butyricimonas	PD and Aging	Mild and moderate (7↑)	More Asian	SCFA (butyrate)	Yes
Bifidobacterium	PD and Aging	Mild and moderate (11↑)	More Western	SCFA (acetate) and lactate	Yes
Lactobacillus	PD and Aging	Mild and moerate (11↑)	More Western	Lactic acid	Yes
Verrucomicrobiaceae	PD and Aging	Mild PD (4↑)	Western	No	
Christensenellaceae	PD and Aging	Mild PD (8↑)			No
Bilophila	PD unique	Mild and moderate (4↑)			No
Lactobacillaceae	PD unique	Mild PD (4↑)	Western	Lactic acid	No
Christensenella	PD unique	Moderate PD (5↑)			No
Lachnospiraceae	PD and Aging	Mild and moderate (11↓)	More Western	SCFA (acetate, propionate, butyrate)	Yes
Faecalibacterium	PD and Aging	Mild and moderate (11↓)	More Western	SCFA (butyrate)	Yes
Roseburia	PD and Aging	Mild and moderate (12↓)	More Western	SCFA (butyrate)	Yes
Ruminococcus	PD and Aging	Mild and. moderate (7↓/2↑)	More Asian	SCFA (butyrate)	Yes
Prevotella	PD and Aging	Mild PD (16↓/3↑)	Common/Western	SCFA (propionate)	Yes
Blautia	PD and Aging	Moderate PD (6↓)	More Western	SCFA (butyric and acetic acid)	Yes
Fusicatenibacter	PD and Aging	Moderate PD (4↓)			Yes
Streptococcus	PD unique	Mild PD (2↓)	Asian	D-lactate	Yes
Prevotellaceae	PD unique	Moderate PD (7↓)			Yes
					Common across ethnicity
					Asian
					Western

### Gut microbiome among PD patients in different ethnicities

In order to evaluate the role of differing ethnicities on the PD gut microbiome, we reviewed original PD articles that were grouped based on ethnicity. The Jaccard Distance is 0.77 for gut microbiome that increased in abundance for both Asian and Western PD populations. Similarly, the Jaccard Distance is 0.85 for gut microbiome that decreased in abundance. This indicates that gut microbiome changes are vastly different in PD populations from different ethnicities. Although minimal, the overlap between the various groups offers an intriguing set of PD microbiome changes that are triangulated from and hence common across differing ethnicities; these PD microbiome changes therefore warrant closer examination. Table 4 shows a summary of the gut microbes that are consistently associated with PD despite ethnicity, along with those that are Asian PD specific (Asian unique complement) or Western PD specific (Western unique complement).

Among papers that studied the Asian population, the top frequently reported gut microbes that were found to be increased are *Alistipes* (50%), *Butyricimonas* (42%), *Parabacteroides* (42%), *Akkermansia* (33%), and *Odoribacter* (33%); whereas for papers reporting on Western populations, the top frequently reported gut microbes that were increased are *Akkermansia* (65%), *Bifidobacterium* (35%), and *Lactobacillus* (35%). Among these, *Akkermansia* was consistently reported to be increased in the microbiome regardless of ethnicity. Interestingly, recent studies suggest that *Akkermansia* may act detrimentally by disrupting the intestinal barrier which subsequently leads to chronic inflammation which could consequently aggravate the development of PD (Heneka et al., 2015; Wang K. et al., 2022; Bellini et al., 2023; Kendall et al., 2025; Pfaffinger et al., 2025); however, it should be noted that different strains of *Akkermansia* may have contradicting effects on intestinal barrier dysfunction with some strains (such as *ATCC BAA-835* and *BCRC 18949*) proving to be more protective than detrimental (Ring et al., 2019; Huang et al., 2024). In addition, the top frequency hits for gut microbes that were increased in the Asian unique complement are *Odoribacter* and *Acinetobacter* while those for the Western unique complement are *Lactobacillaceae* and *Verrucomicrobiaceae*.

On the other hand, the top frequently reported gut microbes that were found to be decreased amongst Asians are *Prevotella* (30%) and *Ruminococcus* (30%); whereas in the Western populations these are *Roseburia* (47%), *Faecalibacterium* (41%), *Lachnospiraceae* (35%), and *Prevotella* (18%). Among these, *Prevotella* was consistently reported amongst the top decreased microbes across ethnicities. *Roseburia, Faecalibacterium* and *Lachnospiraceae* were more commonly reported to be decreased in Western PD population. In addition, the top frequency hits for gut microbiome that decreased in the Asian unique complement is *Streptococcus*, while that for the Western complement is *Fusicatenbacter*.

An obvious reason for the microbiome differences observed with various ethnicities is diet. Asian food tends to be rich in carbohydrate, fiber, antioxidants, vitamins, and minerals while being low in fat (Conteh and Huang, 2020). In contrast, Western food is typically high in fat, sodium, protein, and refined sugars (Martinez-Medina et al., 2014; Conteh and Huang, 2020). Diet is known to profoundly change the gut microbiome (David et al., 2014). A study by Yamashita et al. (2019) showed that *Odoribacter*, a genus that we observed to be increased in the Asian group, decreased in Japanese men who adopted a westernized lifestyle after immigrating (Yamashita et al., 2019). Our observations of *Lactobacillaceae* and *Faecalibacterium* being more prevalent in Western populations were also supported by previous studies that demonstrated a high fat diet increasing the abundance of *Lactobacillus spp*. (Okazaki and Katayama, 2021) and that the gut microenvironment of US children were enriched with *Faecalibacterium* (Shankar et al., 2017). Importantly, both Asian and Western uniquely changed microbiome sets were observed to be related to both mild and moderate PD symptom severity (Table 4), suggesting that there is no ethnicity unique microbe that selects for clinical severity of PD.

### Temporal consistency of aging- and PD-associated gut microbiota highlights likely true microbial targets

The cost of sequencing techniques has dramatically reduced over recent years allowing better sequencing depth, greater accessibility, and consequently more publications (Hung et al., 2022). We subsequently pondered if these recent publications corroborate our findings. Therefore, as a follow up to our initial review of articles from 2017 to 2022, we performed a similar search strategy for gut microbiome related studies published in the recent 3 years, from 2023 to 2025. There were close to 800 articles, more than the 689 articles published from 2017 to 2022, confirming a growing interest in the field. After filtering through the selection criteria for original research and relevance to PD or aging, we further examined another 26 papers on PD (Table 5; Bolliri et al., 2022; Wallen et al., 2022; Babacan Yildiz et al., 2023; Huang et al., 2023; Mehanna et al., 2023; Nuzum et al., 2023; Palacios et al., 2023; Pavan et al., 2023; Zhang L. et al., 2023; Duru et al., 2024; Forero-Rodríguez et al., 2024; Metcalfe-Roach et al., 2024; Park et al., 2024; Stagaman et al., 2024; Yan and Zhao, 2024; Yoon et al., 2024; Zhao et al., 2024; Ilie et al., 2025; Jacob et al., 2025; Liu et al., 2025; Papić et al., 2025; Shalash et al., 2025; Villette et al., 2025a,b; Wang et al., 2025; Zhang et al., 2025) and 8 papers on aging (Table 6; Leite et al., 2022; Liu et al., 2023; Pang et al., 2023; Sun et al., 2023; Chen et al., 2024; Chulenbayeva et al., 2024; Ma et al., 2024; Mohammadzadeh et al., 2025) to identify if the data from recent publications support our findings (Supplementary Tables 1, 2).

**TABLE 5:** Compilation of PD papers analyzed (2023–2025).

No.	Paper	Authorship year	Sample	Type	Control No.	PD no.	Race	Age range	PD staging (score if available)	Data availability
P35	Difference in gut microbial dysbiotic patterns between body-first and brain-first Parkinson’s disease	Park et al., 2024	Fecal	Article	36	36	Japan	40–75	UPDRS III (23)	PRJNA1043247
P36	Differences in the gut microbiome across typical ageing and in Parkinson’s disease	Nuzum et al., 2023	Fecal	Article	55	18	Australia	50–80	UPDRS II (8); H&Y (1)	Upon request
P37	Microbial biomarker discovery in Parkinson’s disease through a network-based approach	Zhao et al., 2024	Fecal	M eta-ana lysis	456	550	Across 4 countries	64–69	UPDRS III (22.9, 27.5, 31.8, 33.8)	PRJEB55464, PRJNA391524, DRA009229, PRJNA381395, PRJEB27564
P38	Metagenomic Analysis Reveals Large-Scale Disruptions of the Gut Microbiome in Parkinson’s Disease	Metcalfe-Roach et al., 2024	Fecal	Article	100	176	Canada	40–85	UPDRS III (21); H&Y (2)	Upon request
P39	Integrated multi-omics highlights alterations of gut microbiome functions in prodromal and idiopathic Parkinson’s disease	Villette et al., 2025b	Fecal	Article	49	46	Luxembourg	60–80	NIL	PRJNA782492
P40	Study of the gut microbiome in Egyptian patients with Parkinson’s Disease	Mehanna et al., 2023	Fecal	Article	35	30	Egypt	60–80	Total UPDRS (46.5); H&Y (mild)	Upon request
P41	Oral and gut microbiome profiles in people with early idiopathic Parkinson’s disease	Stagaman et al., 2024	Fecal	Article	221	445	US	58–66	UPDRS II	Available at FOXDEN(MJFF)
P42	Metagenomics of the Gut Microbiome in Parkinson’s Disease: Prodromal Changes	Palacios et al., 2023	Fecal	Article	131	176	US	79–95	Prodromal and Recently diagnosed	dbGap (phs002193.v1.p1)
P43	Metagenomics of Parkinson’s disease implicates the gut microbiome in multiple disease mechanisms	Wallen et al., 2022	Fecal	Article	234	490	US	50–65	NIL	PRJNA834801
P44	Gut microbiome dysbiosis across early Parkinson’s disease, REM sleep behavior disorder and their first-degree relatives	Huang et al., 2023	Fecal	Article	108	36	China/Hong Kong	60–70	Early PD	PRJEB52086
P45	Human gut microbiome gene co-expression network reveals a loss in taxonomic and functional diversity in Parkinson’s disease	Villette et al., 2025a	Fecal	Article	49	46	Luxembourg	60–80	NIL	PRJNA782492
P46	The Associations Among Gut Microbiota, Branched Chain Amino Acids, and Parkinson’s Disease: Mendelian Randomization Study	Yan and Zhao, 2024	Fecal	Article	Total 7,738		Dutch	NIL	NIL	GCST90027446-GCST90027857
P47	Gut Microbiota in Monozygotic Twins Discordant for Parkinson’s Disease	Bolliri et al., 2022	Fecal	Article	20	20	Italy	57–67	H&Y (2)	Upon request
P48	Gut microbial community of patients with Parkinson’s disease analyzed using metagenome-assembled genomes	Zhang et al., 2025	Fecal	Article	41	81	China	60–68	UPDRS III (33); H&Y (2.3)	SRP515491
P49	Metagenome-assembled microbial genomes from Parkinson’s disease fecal samples	Duru et al., 2024	Fecal	Article	68	68	Europe	60–65	NIL	PRJEB59350
P50	Dietary quality and the gut microbiome in early-stage Parkinson’s disease patients	Yoon et al., 2024	Fecal	Article	81	85	Korea	56–76	H&Y (2.4)	Upon request
P51	Causal Relationship Between Intestinal Microbiota, Inflammatory Cytokines, Peripheral Immune Cells, Plasma Metabolome and Parkinson’s Disease: A Mediation Mendelian Randomization Study	Wang et al., 2025	Fecal	Article	Total 5,959		Finland	NIL	NIL	FINRISK2002
P52	Changes in the intestinal microbiota of patients with Parkinson’s disease and their clinical significance	Zhang L. et al., 2023	Fecal	Article	20	20	China	No full text access		
P53	Changes in Bacterial Gut Composition in Parkinson’s Disease and Their Metabolic Contribution to Disease Development: A Gut Community Reconstruction Approach	Forero-Rodríguez et al., 2024	Fecal	Article	25	25	Colombia	NIL	NIL	PRJNA975118
P54	Exploring the gut microbiota-Parkinson’s disease link: preliminary insights from metagenomics and Mendelian randomization	Liu et al., 2025	Fecal	Article	15	25	Mongolia	58–80	H&Y (1.5)	PRJNA1329258
P55	Exploring gut microbiota alterations in Parkinson’s disease: insights from a 16S amplicon sequencing Eastern European pilot study	Ilie et al., 2025	Fecal	Article	20	19	Eastern Europe	37–89	NIL	https://zenodo.org/records/15647546
P56	Gut microbial shifts toward inflammation in Parkinson’s disease: Insights from pilot shotgun metagenomics Egyptian cohort	Shalash et al., 2025	Fecal	Article	6	7	Egypt	NIL	NIL	Upon request
P57	Gut microbiome differences in Parkinson’s disease patients in Central Kerala population	Jacob et al., 2025	Fecal	Article	16	16	India	NIL	NIL	PRJNA1178079
P58	Microbial diversity in drug-naïve Parkinson’s disease patients	Papić et al., 2025	Fecal	Article	34	49	Croatia	33–74	UPDRS III (21)	PRJNA1196315
P59	Dysbiosis of the Beneficial Gut Bacteria in Patients with Parkinson’s Disease from India	Pavan et al., 2023	Fecal	Article	13	23	India	48–69	UPDRS III (38)	NIL
P60	Altered gut microbiota in patients with idiopathic Parkinson’s disease: an age-sex matched case-control study	Babacan Yildiz et al., 2023	Fecal	Article	42	42	Turkey	No full text access		
										Mild PD
										Moderate PD

**TABLE 6:** Compilation of aging-related papers analyzed (2023–2025).

No.	Paper	Authorship year	Sample	Type	Control (Young) No.	Aged No.	Centenarian No.	Race	Age range	Data availability
AG12	The small bowel microbiome changes significantly with age and aspects of the ageing process	Leite et al., 2022	Small intestinal microbiome	Article	Total 251		0	Us	18–35; 36–50; 51–65; 66–80	NIL
AG13	Longevity of centenarians is reflected by the gut microbiome with youth-associated signatures	Pang et al., 2023	Fecal	Article	314	386	297	China	20–44; 66–85; 100–117	PRJNA830660
AG 14	Age-dependent changes in the gut microbiota and serum metabolome correlate with renal function and human aging	Sun et al., 2023	Fecal	Article	35	87	29	China	20–60; 60–100; 100–111	CNP0000634
AG15	Age-related dynamics of predominant methanogenic archaea in the human gut microbiome	Mohammadzadeh et al., 2025	Fecal	Article	127	86	34	Austria	19–59; 60–99; 100–109	PRJEB72212
AG16	Mendelian randomization analyses reveal causal relationships between the human microbiome and longevity	Liu et al., 2023	Fecal, Oral	Article	Total 1,539			China	Not included	CNP0000794
AG17	The Trajectory of Successful Aging: Insights from Metagenome and Cytokine Profiling	Chulenbayeva et al., 2024	Fecal	Article	31		46	Kazakhstan	35–48; 93–103	PRJNA973824
AG18	Comprehensive gut microbiota composition and microbial interactions among the three age groups	Ma et al., 2024	Fecal	Article	99	177	270	Italy, Japan, China	21–55; 65–89; 90–109	Italy: PRJEB25514 and PRJNA553191; China: PRJNA624763; Japan: PRJNA675598
AG19	Consistent signatures in the human gut microbiome of longevous populations	Chen et al., 2024	Fecal	Article	148	574	434	Italy, Japan, China	Combination of different studies	CNP0004699 and CNP0005686

The top 10 most commonly reported gut microbiome changes for both PD and aging are collated in Table 7 (*↑: increased abundance in both PD and aging, *↓: decreased abundance in both PD and aging). A comparison between the time periods of 2017–2022 (Table 3) and 2023–2025 (Table 7) revealed consistency in the top commonly reported gut microbes, with the Jaccard Index averaging at 0.23. *Lactobacillus, Akkermansia, Bifidobacterium*, and *Alistipes* are consistently reported as increased in PD while *Roseburia, Faecalibacterium, Blautia*, and *Fusicatenibacter* are consistently reported as decreased in PD regardless of the time period. Similarly, *Alistipes, Akkermansia*, and *Parabacteriods* are consistently reported as increased in aging populations while *Lachnospiraceae, Faecalibacterium, Blautia* and *Dorea* are consistently reported as decreased in aging across both time periods. These observations confirm that there was temporal consistency which indicates that our findings are temporally robust. Additionally, further analysis examining PD and aging cohorts across both time periods revealed that *Akkermansia* and *Alistipes* are frequently increased in abundance while *Faecalibacterium, Blautia*, and *Fusicatenibacter* are frequently decreased in abundance (Table 7); the temporally consistent association of these microbes with both PD and aging highlights their importance as likely true microbial targets (i.e., reliable microbial patterns of PD and aging).

**TABLE 7:** Top 10 gut microbiome changes in PD and aging (2023–2025) and comparison with Table 3.

Microbiome	%Up PD	Microbiome	%Down PD	Microbiome	%Up Aging	Microbiome	%Down Aging
Lactobacillus	20.000	Roseburia	28.000	Alistipes *↑	37.500	Lachnospiraceae	50.000
Akkermansia * ↑	16.000	Faecalibacterium *↓	24.000	Akkermansia *↑	25.000	Faecalibacterium *↓	37.500
Bifidobacterium	16.000	Blautia *↓	20.000	Clostridia	25.000	Faecalibacterium prausnitzii *↓	37.500
Bifidobacterium dentium	12.000	Faecalibacterium prausnitzii *↓	20.000	Clostridiaceae	25.000	Anaerostipes hadrus	25.000
Bifidobacterium longum	12.000	Fusicatenibacter *↓	16.000	Enterobacteriaceae	25.000	Blautia *↓	25.000
Collinsella	12.000	Butyricicoccus	12.000	Escherichia coli	25.000	Clostridium	25.000
Streptococcus	12.000	Bifidobacterium adolescentis	8.000	Methanobrevibacter smithii	25.000	Dorea	25.000
Alistipes *↑	8.000	Blautia wexlerae	8.000	Parabacteroides	25.000	Fusicatenibacter *↓	25.000
Alistipes indistinctus	8.000	Eubacterium eligens	8.000	Proteobacteria	25.000	XanthomonadaceaeÂ	25.000
Bacteroides intestinalis	8.000	Eubacterium rectale	8.000	ActinobacillusÂ	12.500	Acinetobacter	12.500
Consistently in the top 10 across both time periods.							

*↑ Up in both PD and aging. *↓ Down in both PD and aging.

We next assessed whether the gut microbes that were consistently associated with PD across ethnicities (Table 4) remain relevant across both time periods. Notably, 76% of the microbes listed in Table 4 continue to be reported, and all are short-chain fatty acid (SCFA) or butyrate producers, suggesting that butyrate may be a key metabolite.

### Aging/PD-related gut microbiome may exert effects via a common butyrate metabolite

Metabolites derived from microbes have an impact on the development of brain dysfunction (Banfi et al., 2021). As such, we mapped the top metabolite associated with given microbes (Table 4; Feiner, 2006; Biddle et al., 2013; Morrison and Preston, 2016; Walker et al., 2017; Ozato et al., 2019; Vitetta et al., 2019; Parker et al., 2020; Kelly et al., 2021; Lei et al., 2021; Li Z. et al., 2021; Oh et al., 2021; Ren et al., 2021; Lee et al., 2022). Examination of some of the ethnic-specific microbes, such as *Alistipes* and *Odoribacter* in Asians and *Lactobacillus* in Westerners, yield interesting findings. Specifically, *Alistipes* and *Odoribacter* are linked to the production of sulfonoliplid (Walker et al., 2017; Parker et al., 2020), which can increase the expression of pro-inflammatory cytokines such as IL-1α, IL-1β, IL-6, and TNFα (Hou et al., 2022) that are commonly implicated in PD (Nagatsu and Sawada, 2005). Given that the abundance of *Alistipes* and *Odoribacter* also increases with aging (Table 4), increased inflammation from sulfonolipid might be an avenue through which aging exerts its impact on PD risk. Similarly, *Lactobacillus* is linked to the production of lactic acid (Feiner, 2006). High levels of lactate have been reported in brain regions of PD patients (Ding et al., 2022), potentially first produced in the gut and crossing the blood brain barrier (Knudsen et al., 1991) as a form of coping mechanism against PD (Adams, 2021).

Despite the diverse pool of gut microbiome changes in both aging and PD, the microbiome changes converge on a common metabolite—butyrate (Table 4). Butyrate is a short chain fatty acid that is produced by anaerobic fermentation of dietary fiber and is one of the energy sources for colonic epithelial cells (Cantu-Jungles et al., 2019; Fock and Parnova, 2023). In addition to regulating gut health, it has been shown to modulate brain function (Cantu-Jungles et al., 2019; Getachew et al., 2020). Butyrate could exert its effects by acting as a strong histone deacetylation inhibitor which affects epigenetics (Candido et al., 1978). In support of this, a recent study demonstrated the link between reduced gut butyrate levels with epigenetic changes observed in PD neutrophils and neurons; many of the butyrate-associated methylation sites overlap with risk genes involving PD (Xie et al., 2022). In previous studies, gut microbiome from PD patients was shown (via *in vitro* fecal fiber fermentation) to have reduced capability of producing butyrate compared to healthy controls (Baert et al., 2021). The observation is likely due to the decreased abundance of the main butyrate-producing microbes *Faecalibacterium, Ruminococcus* and *Roseburia* (Morrison and Preston, 2016) in agreement with our analysis. Interestingly, butyrate production decreases with age as *Faecalibacterium* and *Ruminococcus* are aging-PD confounded; this may be one of the mechanisms through which aging exerts its effect on PD risk. Additionally, since butyrate acts an energy source for intestinal epithelial cells, it has a crucial role to play in the maintenance of the intestinal barrier and gut permeability (Jobin, 2014; Getachew et al., 2020; Karunaratne et al., 2020). This is noteworthy because under certain microbial dysbiosis conditions, intestinal inflammation may detrimentally increase gut permeability allowing inflammatory bacterial metabolites such as lipopolysaccharides (LPS), and inflammatory cytokines such as IL-1α, IL-1β, IL-6, and TNFα to escape into the bloodstream; eventually these could penetrate the blood brain barrier and exacerbate neuroinflammation culminating in dopaminergic neuronal death and eventually PD development (Wang et al., 2021; Chidambaram et al., 2022; Zhu et al., 2022; Guo et al., 2023). Therefore, butyrate’s role in maintaining a healthy intestinal barrier could theoretically impede this harmful process, allowing it to be neuroprotective.

There is currently no strong evidence suggesting that decreased abundance of butyrate-producing microbes (and consequent butyrate depletion) has a causal relationship with PD development, and no clinical trials have proven that butyrate-deficiency leads to PD. However, it is interesting to note that butyrate loss has been linked with constipation, a well-established prodromal PD symptom (Cirstea et al., 2020; Aho et al., 2021; Tan et al., 2021; Yuan et al., 2024) that has been shown to precede overt motor deficits in PD patients by years (Ross et al., 2012). Here, butyrate exerts its beneficial effects by improving gastrointestinal motility (Fukumoto et al., 2003; Vincent et al., 2018; Yuan et al., 2024) which subsequently reduces constipation severity (Tan et al., 2021; Yuan et al., 2024).

## Increasing evidence on association of decreased butyrate with PD and pre-clinical treatment models

In the last 5 years (2021–2025), more population studies have emerged identifying decreased butyrate as a biomarker for PD (Yan et al., 2022; Liu et al., 2024; Zhao et al., 2024; Rust et al., 2025). In addition to constipation, studies suggest butyrate plays an early role in other prodromal PD non-motor symptoms such as REM sleep disorder and depression; butyrate-producing bacteria such as *Lachnospira*, *Butyricicoccus*, and *[Eubacterium]_ventriosum_group* where found to be decreased in REM sleep disorder (Huang et al., 2023), and likewise butyrate-producing *Roseburia* and *Romboutsia* were observed to be reduced in depression (Xie et al., 2022). Even within the PD group, lower abundance of butyrate producing *Butyricimonas synergistica*, was associated with worse non-motor symptoms (Nuzum et al., 2023). Other studies have suggested that the reduction of fecal butyrate correlated with clinical severity of PD (Chen et al., 2022), such as worse postural instability-gait disorder scores (Tan et al., 2021). Interestingly, in a human A53T α-synuclein transgenic mouse model, enteric α-synuclein expression was shown to decrease fecal butyrate levels (Pellegrini et al., 2022). Furthermore, *in vitro* fermentation experiments using fecal samples from PD patients and age-matched healthy controls demonstrated that, although butyrate production can be stimulated in PD patients, overall butyrate production rate remain significantly lower (Baert et al., 2021). Taken together, these findings suggest that PD-initiating factors such as α-synuclein may contribute to an early reduction in butyrate levels, which are subsequently maintained at low levels throughout disease progression due to impaired production in PD patients. This highlights butyrate as a promising biomarker for PD.

A natural question that follows is whether changing the gut microbiome and more specifically supplementation of butyrate can then improve PD symptoms. In a recent study, fecal microbiota transplantation (FMT) from healthy human individuals to 1-methyl-4-phenyl-1,2,3,6-tetrahydropyridine (MPTP)-induced PD mouse models was able to significantly improve motor function, with the therapeutic effects associated with increased levels of butyrate (Ni et al., 2025). Importantly, supplementation of butyrate alone (in the form of sodium butyrate) was sufficient to reduce motor deficits in an α-synuclein pre-formed fibrils (PFF) mouse model (Kakoty et al., 2021), improve non-motor symptoms like anxiety in 6-hydroxydopamine (6-OHDA) mice (Avagliano et al., 2025), and normalize sleep architecture in MPTP mice (Duan et al., 2025).

Several studies suggest that a key mechanism underlying the beneficial properties of butyrate is through re-shaping the gut microbiota and reducing colonic and intestinal barrier disruption (Avagliano et al., 2022; Xu et al., 2022; Guo et al., 2023; Zhang Y. et al., 2023) across various neurotoxin-induced mouse models of PD involving rotenone (Zhang Y. et al., 2023), 6-OHDA (Avagliano et al., 2022) or MPTP (Xu et al., 2022; Guo et al., 2023). These studies also reported another key mechanism of reduced gut and brain inflammation. Supporting this, sodium butyrate was shown to be able to suppress MPP+ activation of BV2 microglia cells and reduce the production of nitrite and pro-inflammatory cytokines (Xu et al., 2024). In a separate study, butyrate attenuated 6-OHDA-induced dopaminergic neuronal injury via inhibiting microglia activation and neuroinflammatory factors production (Xu et al., 2025). Other pathways suggested includes PGC1α-autophagy activation to reduce rotenone-induced α-synuclein accumulation and aggregation (Zhang Y. et al., 2022), inhibition of pro-inflammatory pathways involving JAK2/STAT3 signaling (Ji et al., 2023), NF-κB and MAPK signaling in the SNpc (Hou et al., 2022), and protection against Fe and Mn toxicities (Tizabi et al., 2023). Additionally, there are also suggestions to supplement L-Dopa treatment with butyrate as HNK-butyrate esters have been shown to inhibit *E. faecalis* growth in the gut, thus increasing the amount of unmetabolized L-Dopa that can reach the brain for therapeutic effects (Cheng et al., 2025).

While butyrate shows therapeutic promise, its efficient delivery remains an important point of consideration. As with other SCFAs, butyrate is easily absorbed in the human small intestine (specifically the jejunum) which limits butyrate bioavailability in the lower gastrointestinal tract such as the colon (Schmitt et al., 1976; Hodgkinson et al., 2023). There are currently several ways of butyrate delivery that aim to overcome this issue. The first technique is to provide butyrate as a triglyceride. Butyrate triglyceride is composed of 3 butyric acid molecules connected to a glycerol backbone; importantly, this conformation prevents premature absorption of butyrate in the small intestine (Bedford and Gong, 2018). Based on this principle, a retrospective clinical study examining existing medical records of PD patients showed that combination probiotics supplementation of butyrate triglyceride (302.86 mg) with Crocus sativus L. (30 mg) and vitamin D3 (100 mcg) was sufficient to improve UPDRS III scores (Alexoudi et al., 2023). Secondly, a similar technique of butyrate delivery involves the use of microencapsulated sodium butyrate; this colonic-release butyrate capsules have been shown to be effective in alleviating symptoms of irritable bowel syndrome (IBS) (Banasiewicz et al., 2013), ulcerative colitis (UC) (Vernero et al., 2020), and symptomatic uncomplicated diverticular disease (SUDD) (Tursi et al., 2025). Thirdly, butyrate levels could be improved by administering prebiotics such as resistant starch. Prebiotic resistant starch is able to pass through the small intestine intact and reach the colon where it can then aid butyrate production (through microbial fermentation) (Dobranowski and Stintzi, 2021; Hodgkinson et al., 2023). Recently, a clinical trial aimed at altering fecal SCFAs used an 8-week resistant starch prebiotic intervention to significantly increase fecal butyrate concentrations (Becker et al., 2022) in PD patients. Lastly, FMT could be used to deliver healthy butyrate-producing bacteria directly to the colon. A 2017 randomized controlled trial showed that UC patients who responded clinically to FMT treatment had more butyrate-producing bacteria post-FMT (Fuentes et al., 2017). In the context of PD however, FMT has been less successful in improving clinical outcomes in patients (Scheperjans et al., 2024). Nevertheless, FMT may perform better in the case of prodromal PD. By the time a PD patient is clinically diagnosed more than 50% of nigrostriatal dopaminergic neurons have already been lost (DeMaagd and Philip, 2015); this could potentially limit how effective FMT could be at this clinical stage. Therefore, the timing of FMT intervention may play an important role in overall success. All in all, butyrate shows promise as a biomarker and a feasible therapeutic target for PD.

## Discussion/Conclusion

Current studies tend to examine aging and Parkinson’s disease separately. In this review, we have examined gut microbiome changes that relate to both aging and PD. We have also examined the gut microbiome differences that are related to ethnicity and identified several microbes that were ethnically specific: *Alistipes* and *Odoribacter* for Asians, *Lactobacillus* and *Roseburia* for Western populations. Importantly, we observed that the gut microbiome seemed to converge by exerting its effects through butyrate. Notably, the major producers of butyrate—*Faecalibacterium* and *Ruminococcus*—decreased in abundance across both Asian and Western populations as well as with age. One possible reason for this observation could be the beneficial roles played by butyrate as a histone deacetylation inhibitor and an important contributor to intestinal barrier and gut permeability. Our findings are reaffirmed by a recently published metagenomics by Wallen et al. (2022) on the largest PD cohort of microbiome data, which determined that the abundance of bacteria such as *Blautia, Faecalibacterium, Fusicatenibacter, Roseburia* and *Ruminococcus* were decreased in PD, while *Bifidobacterium* and *Lactobacillus* were increased in PD (Wallen et al., 2022). Additionally, we observed bidirectional changes for *Prevotella* that was likewise pointed out by Wallen et al and resolved to be generally upregulated in their meta-analysis; the study also discussed the observed increases in *Akkermansia* in PD that was not detected in the metagenomics (Wallen et al., 2022). Many of these microbes along with butyrate are altered with age suggesting that these may be early targets for preventive measures against PD.

In recent years, several papers have stratified their PD cohorts to examine the effect of medication as a confounder on gut microbiome (Hill-Burns et al., 2017; Palacios et al., 2021; Lubomski et al., 2022b; Gorecka-Mazur et al., 2024). Only patients who were on Levodopa-Carbidopa and Deep Brain Stimulation (DBS) (Palacios et al., 2021; Lubomski et al., 2022b) reflected changes in gut microbiome but not those on COMT inhibitors (Hill-Burns et al., 2017). Interestingly, DBS and Levodopa affect different microbiomes, with the common downregulated microbes being *Hespellia* and the common upregulated microbes being *Prevotella* and *Bacillus* (Lubomski et al., 2022b). The sensitivity of *Prevotella* to PD treatments may partly explain the bidirectional changes in this genus reported across studies. Furthermore, levodopa-associated microbiome changes emerged only at 6 months (Lubomski et al., 2022b) and not at the earlier 3-month time point (Palacios et al., 2021), suggesting a delayed or cumulative treatment effect. While *Roseburia*, a key butyrate-producing genus, was upregulated following prolonged levodopa exposure, most taxa that increased across treatment intervals (including *Prevotella*, *Bacillus*, *Methanobrevibacter*, and *Veillonella*) are not direct butyrate producers and are more commonly associated with acetate, succinate, propionate production, or methanogenesis. Importantly, these medication-associated microbiome changes were distinct from the core microbial signatures identified as PD- or aging-related, underscoring the need to account for treatment effects when interpreting disease-associated gut microbiome alterations.

This study has several limitations. Firstly, only two major demographic populations (Asian and Western) were included in the analysis which limits how well the findings extrapolate on a global scale, especially since the gut microbiome is sensitive to factors such as dietary pattern and geographical location (Leeming et al., 2019). This limitation is due to the lack of literature studying certain demographics, particularly from developing nations which may lack the required resources, and highlights the need for more thorough demographic representation in the field. Secondly, this review primarily reports on 16S rRNA sequencing data. While this approach allows quantification of bacterial composition, it fails to capture any information on the other microorganisms that make up the gut microbiome (e.g., fungi and viruses) (Bars-Cortina et al., 2024). Additionally, 16S rRNA data does not account for functional activity (Durazzi et al., 2021). Although more costly, newer techniques such as metagenomic sequencing address these limitations while offering better taxonomic resolution (Kuczynski et al., 2012; Durazzi et al., 2021). Thirdly, none of the studies examined in this review reported on the gut microbiome of severely symptomatic PD patients (UPDRS III above 58) who may present different gut microbial signatures than mildly (UPDRS III below 33) and moderately symptomatic PD (UPDRS III between 33 and 58) (Martínez-Martín et al., 2015); therefore, although we show that gut microbes that are associated with both aging and PD did not appear to correlate with mild and moderately symptomatic PD, caution should be used when extrapolating these findings to more symptomatic PD patients.

## References

[B1] Adams J. (2021). Possible causes of Parkinson’s disease. *Front. Biosci.* 26:387–394. 10.52586/4952 34455768

[B2] Aho V. Houser M. Pereira P. Chang J. Rudi K. Paulin L. et al. (2021). Relationships of gut microbiota, short-chain fatty acids, inflammation, and the gut barrier in Parkinson’s disease. *Mol. Neurodegener.* 16:6. 10.1186/s13024-021-00427-6 33557896 PMC7869249

[B3] Aho V. Pereira P. Voutilainen S. Paulin L. Pekkonen E. Auvinen P. et al. (2019). Gut microbiota in Parkinson’s disease: Temporal stability and relations to disease progression. *EBioMedicine* 44 691–707. 10.1016/j.ebiom.2019.05.064 31221587 PMC6606744

[B4] Alafifi T. Bakhsh A. Elbashari M. Abouelnaga M. Eldimllawi A. M. (2020). A novel mutation of PARK-2 gene in a patient with early-onset Parkinson’s disease. *Oman Med. J.* 35:e140. 10.5001/omj.2020.58 32647592 PMC7335453

[B5] Alexoudi A. Kesidou L. Gatzonis S. Charalampopoulos C. Tsoga A. (2023). Effectiveness of the combination of probiotic supplementation on motor symptoms and constipation in Parkinson’s disease. *Cureus* 15:e49320. 10.7759/cureus.49320 38146566 PMC10749423

[B6] Avagliano C. Coretti L. Lama A. Pirozzi C. De Caro C. De Biase D. et al. (2022). Dual-Hit model of Parkinson’s disease: Impact of dysbiosis on 6-hydroxydopamine-insulted mice-neuroprotective and anti-inflammatory effects of butyrate. *Int. J. Mol. Sci.* 23:6367. 10.3390/ijms23126367 35742813 PMC9223521

[B7] Avagliano C. De Caro C. Cuozzo M. Roberti R. Russo E. La Rana G. et al. (2025). Sodium Butyrate ameliorates pain and mood disorders in a mouse model of Parkinson disease. *Biomed. Pharmacother.* 184:117903. 10.1016/j.biopha.2025.117903 39938349

[B8] Babacan Yildiz G. Kayacan Z. Karacan I. Sumbul B. Elibol B. Gelisin O. et al. (2023). Altered gut microbiota in patients with idiopathic Parkinson’s disease: An age-sex matched case-control study. *Acta Neurol. Belg.* 123 999–1009. 10.1007/s13760-023-02195-0 36719617

[B9] Badal V. Vaccariello, Murray E. Yu K. Knight R. Jeste D. et al. (2020). The Gut microbiome, aging, and longevity: A systematic review. *Nutrients* 12:3759. 10.3390/nu12123759 33297486 PMC7762384

[B10] Baert F. Matthys C. Maselyne J. Van Poucke C. Van Coillie E. Bergmans B. et al. (2021). Parkinson’s disease patients’ short chain fatty acids production capacity after in vitro fecal fiber fermentation. *NPJ Parkinsons Dis.* 7:72. 10.1038/s41531-021-00215-5 34389734 PMC8363715

[B11] Baldini F. Hertel J. Sandt E. Thinnes C. C. Neuberger-Castillo L. Pavelka L. et al. (2020). Parkinson’s disease-associated alterations of the gut microbiome predict disease-relevant changes in metabolic functions. *BMC Biol.* 18:62. 10.1186/s12915-020-00775-7 32517799 PMC7285525

[B12] Banasiewicz T. Krokowicz Ł Stojcev Z. Kaczmarek B. F. Kaczmarek E. Maik J. et al. (2013). Microencapsulated sodium butyrate reduces the frequency of abdominal pain in patients with irritable bowel syndrome. *Colorectal Dis.* 15 204–209. 10.1111/j.1463-1318.2012.03152.x 22738315

[B13] Banfi D. Moro E. Bosi A. Bistoletti M. Cerantola S. Crema F. et al. (2021). Impact of microbial metabolites on microbiota-gut-brain axis in inflammatory bowel disease. *Int. J. Mol. Sci.* 22:1623. 10.3390/ijms22041623 33562721 PMC7915037

[B14] Barichella M. Severgnini M. Cilia R. Cassani E. Bolliri C. Caronni S. et al. (2019). Unraveling gut microbiota in Parkinson’s disease and atypical parkinsonism. *Mov. Disord.* 34 396–405. 10.1002/mds.27581 30576008

[B15] Bars-Cortina D. Ramon E. Rius-Sansalvador B. Guinó E. Garcia-Serrano A. Mach N. et al. (2024). Comparison between 16S rRNA and shotgun sequencing in colorectal cancer, advanced colorectal lesions, and healthy human gut microbiota. *BMC Genomics* 25:730. 10.1186/s12864-024-10621-7 39075388 PMC11285316

[B16] Becker A. Schmartz G. Gröger L. Grammes N. Galata V. Philippeit H. et al. (2022). Effects of resistant starch on symptoms, fecal markers, and gut microbiota in Parkinson’s disease - The RESISTA-PD trial. *Genomics Proteomics Bioinformatics* 20 274–287. 10.1016/j.gpb.2021.08.009 34839011 PMC9684155

[B17] Bedarf J. Hildebrand F. Coelho L. Sunagawa S. Bahram M. Goeser F. et al. (2017). Functional implications of microbial and viral gut metagenome changes in early stage L-DOPA-naïve Parkinson’s disease patients. *Genome Med.* 9:39. 10.1186/s13073-017-0428-y 28449715 PMC5408370

[B18] Bedarf J. Hildebrand F. Goeser F. Bork P. Wüllner U. (2019). [The gut microbiome in Parkinson’s disease]. *Nervenarzt* 90 160–166. 10.1007/s00115-018-0601-6 30171304

[B19] Bedford A. Gong J. (2018). Implications of butyrate and its derivatives for gut health and animal production. *Anim. Nutr.* 4 151–159. 10.1016/j.aninu.2017.08.010 30140754 PMC6104520

[B20] Bellini G. Benvenuti L. Ippolito C. Frosini D. Segnani C. Rettura F. et al. (2023). Intestinal histomorphological and molecular alterations in patients with Parkinson’s disease. *Eur. J. Neurol.* 30 3440–3450. 10.1111/ene.15607 36263629

[B21] Biddle A. Stewart L. Blanchard J. Leschine S. (2013). Untangling the genetic basis of fibrolytic specialization by lachnospiraceae and ruminococcaceae in diverse gut communities. *Diversity* 5 627–640. 10.3390/d5030627

[B22] Billingsley K. Bandres-Ciga S. Saez-Atienzar S. Singleton A. (2018). Genetic risk factors in Parkinson’s disease. *Cell Tissue Res.* 373 9–20. 10.1007/s00441-018-2817-y 29536161 PMC6201690

[B23] Boertien J. Murtomäki K. Pereira P. van der Zee S. Mertsalmi T. Levo R. et al. (2022). Fecal microbiome alterations in treatment-naive de novo Parkinson’s disease. *NPJ Parkinsons Dis.* 8:129. 10.1038/s41531-022-00395-8 36216843 PMC9551094

[B24] Bolliri C. Fontana A. Cereda E. Barichella M. Cilia R. Ferri V. et al. (2022). Gut microbiota in monozygotic twins discordant for Parkinson’s disease. *Ann. Neurol.* 92 631–636. 10.1002/ana.26454 35852145

[B25] Borghammer P. Van Den Berge N. (2019). Brain-first versus gut-first Parkinson’s disease: A hypothesis. *J. Parkinsons Dis.* 9 S281–S295. 10.3233/JPD-191721 31498132 PMC6839496

[B26] Bower J. Maraganore D. Peterson B. McDonnell S. Ahlskog J. Rocca W. (2003). Head trauma preceding PD: A case-control study. *Neurology* 60 1610–1615. 10.1212/01.wnl.0000068008.78394.2c 12771250

[B27] Buchman A. Shulman J. Nag S. Leurgans S. Arnold S. Morris M. et al. (2012). Nigral pathology and parkinsonian signs in elders without Parkinson disease. *Ann. Neurol.* 71 258–266. 10.1002/ana.22588 22367997 PMC3367476

[B28] Candido E. Reeves R. Davie J. (1978). Sodium butyrate inhibits histone deacetylation in cultured cells. *Cell* 14 105–113. 10.1016/0092-8674(78)90305-7 667927

[B29] Cantu-Jungles T. Rasmussen H. Hamaker B. (2019). Potential of prebiotic butyrogenic fibers in Parkinson’s disease. *Front. Neurol.* 10:663. 10.3389/fneur.2019.00663 31281287 PMC6595503

[B30] Chen J. Zhu L. Wang F. Zhu Y. Chen J. Liang C. et al. (2025). Plasma metabolites as mediators between gut microbiota and Parkinson’s disease: Insights from mendelian randomization. *Mol. Neurobiol.* 62 7945–7956. 10.1007/s12035-025-04765-0 39962023

[B31] Chen S. Chen C. Liao H. Lin Y. Wu Y. Liou J. et al. (2022). Association of fecal and plasma levels of short-chain fatty acids with gut microbiota and clinical severity in patients with Parkinson disease. *Neurology* 98 e848–e858. 10.1212/WNL.0000000000013225 34996879 PMC8883514

[B32] Chen S. Zhang Z. Liu S. Chen T. Lu Z. Zhao W. et al. (2024). Consistent signatures in the human gut microbiome of longevous populations. *Gut Microbes* 16:2393756. 10.1080/19490976.2024.2393756 39197040 PMC11364081

[B33] Cheng G. Hardy M. Feix J. Kalyanaraman B. (2025). Inhibition of levodopa metabolism to dopamine by honokiol short-chain fatty acid derivatives may enhance therapeutic efficacy in Parkinson’s disease. *Sci. Rep.* 15:20004. 10.1038/s41598-025-05072-3 40481059 PMC12144245

[B34] Chidambaram S. Essa M. Rathipriya A. Bishir M. Ray B. Mahalakshmi A. et al. (2022). Gut dysbiosis, defective autophagy and altered immune responses in neurodegenerative diseases: Tales of a vicious cycle. *Pharmacol. Ther.* 231:107988. 10.1016/j.pharmthera.2021.107988 34536490

[B35] Chulenbayeva L. Ganzhula Y. Kozhakhmetov S. Jarmukhanov Z. Nurgaziyev M. Nurgozhina A. et al. (2024). The trajectory of successful aging: Insights from metagenome and cytokine profiling. *Gerontology* 70 390–407. 10.1159/000536082 38246133 PMC11008724

[B36] Cirstea M. Yu A. Golz E. Sundvick K. Kliger D. Radisavljevic N. et al. (2020). Microbiota Composition and metabolism are associated with gut function in Parkinson’s disease. *Mov. Disord.* 35 1208–1217. 10.1002/mds.28052 32357258

[B37] Collier T. Kanaan N. Kordower J. (2011). Ageing as a primary risk factor for Parkinson’s disease: Evidence from studies of non-human primates. *Nat. Rev. Neurosci.* 12 359–366. 10.1038/nrn3039 21587290 PMC3387674

[B38] Conteh A. Huang R. (2020). Targeting the gut microbiota by Asian and Western dietary constituents: A new avenue for diabetes. *Toxicol. Res.* 9 569–577. 10.1093/toxres/tfaa065 32905261 PMC7467239

[B39] David L. Maurice C. Carmody R. Gootenberg D. Button J. Wolfe B. et al. (2014). Diet rapidly and reproducibly alters the human gut microbiome. *Nature* 505 559–563. 10.1038/nature12820 24336217 PMC3957428

[B40] De Sciscio M. Bryant R. Haylock-Jacobs S. Day A. Pitchers W. Iansek R. et al. (2025). Faecal microbiota transplant in Parkinson’s disease: Pilot study to establish safety tolerability. *NPJ Parkinsons Dis.* 11:203. 10.1038/s41531-025-01061-5 40634307 PMC12241514

[B41] DeMaagd G. Philip A. (2015). Parkinson’s disease and its management: Part 1: Disease entity, risk factors, pathophysiology, clinical presentation, and diagnosis. *P T* 40 504–532. 26236139 PMC4517533

[B42] Ding Y. Korchak S. Mamone S. Jagtap A. P. Stevanato G. Sternkopf S. et al. (2022). Rapidly signal-enhanced metabolites for atomic scale monitoring of living cells with magnetic resonance. *Chemistry–Methods* 2:e202200023. 10.1002/cmtd.202200023

[B43] Dobranowski P. Stintzi A. (2021). Resistant starch, microbiome, and precision modulation. *Gut Microbes* 13:1926842. 10.1080/19490976.2021.1926842 34275431 PMC8288039

[B44] Dorsey E. Zafar M. Lettenberger S. Pawlik M. Kinel D. Frissen M. et al. (2023). Trichloroethylene: An invisible cause of Parkinson’s disease? *J. Parkinsons Dis.* 13 203–218. 10.3233/JPD-225047 36938742 PMC10041423

[B45] Duan W. Xie W. Ying C. Fen W. Cheng X. Mao C. et al. (2025). Butyrate improves abnormal sleep architecture in a Parkinson’s disease mouse model via BDNF/TrkB signaling. *NPJ Parkinsons Dis.* 11:175. 10.1038/s41531-025-01029-5 40537481 PMC12179271

[B46] Durazzi F. Sala C. Castellani G. Manfreda G. Remondini D. De Cesare A. (2021). Comparison between 16S rRNA and shotgun sequencing data for the taxonomic characterization of the gut microbiota. *Sci. Rep.* 11:3030. 10.1038/s41598-021-82726-y 33542369 PMC7862389

[B47] Duru I. Lecomte A. Shishido T. Laine P. Suppula J. Paulin L. et al. (2024). Metagenome-assembled microbial genomes from Parkinson’s disease fecal samples. *Sci. Rep.* 14:18906. 10.1038/s41598-024-69742-4 39143178 PMC11324757

[B48] Elfil M. Kamel S. Kandil M. Koo B. Schaefer S. (2020). Implications of the Gut Microbiome in Parkinson’s Disease. *Mov. Disord.* 35 921–933. 10.1002/mds.28004 32092186

[B49] Feiner G. (2006). *39 - The Microbiology of Specific Bacteria. Meat Products Handbook.* Delhi: Woodhead Publishing, 595–615.

[B50] Fock E. Parnova R. (2023). Mechanisms of blood-brain barrier protection by microbiota-derived short-chain fatty acids. *Cells* 12:657. 10.3390/cells12040657 36831324 PMC9954192

[B51] Forero-Rodríguez J. Zimmermann J. Taubenheim J. Arias-Rodríguez N. Caicedo-Narvaez J. Best L. et al. (2024). Changes in bacterial gut composition in Parkinson’s disease and their metabolic contribution to disease development: A gut community reconstruction approach. *Microorganisms* 12:325. 10.3390/microorganisms12020325 38399728 PMC10893096

[B52] Fuentes S. Rossen N. van der Spek M. Hartman J. Huuskonen L. Korpela K. et al. (2017). Microbial shifts and signatures of long-term remission in ulcerative colitis after faecal microbiota transplantation. *ISME J.* 11 1877–1889. 10.1038/ismej.2017.44 28398347 PMC5520032

[B53] Fukumoto S. Tatewaki M. Yamada T. Fujimiya M. Mantyh C. Voss M. et al. (2003). Short-chain fatty acids stimulate colonic transit via intraluminal 5-HT release in rats. *Am. J. Physiol. Regul. Integr. Comp. Physiol.* 284 R1269–R1276. 10.1152/ajpregu.00442.2002 12676748

[B54] Getachew B. Csoka A. Bhatti A. Copeland R. Tizabi Y. (2020). Butyrate protects against salsolinol-induced toxicity in SH-SY5Y cells: Implication for Parkinson’s disease. *Neurotox Res.* 38 596–602. 10.1007/s12640-020-00238-5 32572814 PMC7484007

[B55] Goetz C. Tilley B. Shaftman S. Stebbins G. Fahn S. Martinez-Martin P. et al. (2008). Movement disorder society-sponsored revision of the unified Parkinson’s disease rating scale (MDS-UPDRS): Scale presentation and clinimetric testing results. *Mov. Disord.* 23 2129–2170. 10.1002/mds.22340 19025984

[B56] Gorecka-Mazur A. Krygowska-Wajs A. Furgala A. Li J. Misselwitz B. Pietraszko W. et al. (2024). Associations between gut microbiota characteristics and non-motor symptoms following pharmacological and surgical treatments in Parkinson’s disease patients. *Neurogastroenterol. Motil.* 36:e14846. 10.1111/nmo.14846 38873926

[B57] Guo T. Zhang Z. Sun Y. Zhu R. Wang F. Ma L. et al. (2023). Neuroprotective Effects of sodium butyrate by restoring gut microbiota and inhibiting TLR4 signaling in mice with MPTP-induced Parkinson’s disease. *Nutrients* 15:930. 10.3390/nu15040930 36839287 PMC9960062

[B58] Haehner A. Hummel T. Reichmann H. (2011). Olfactory loss in Parkinson’s disease. *Parkinsons Dis.* 2011:450939. 10.4061/2011/450939 21687752 PMC3109349

[B59] Heintz-Buschart A. Pandey U. Wicke T. Sixel-Döring F. Janzen A. Sittig-Wiegand E. et al. (2018). The nasal and gut microbiome in Parkinson’s disease and idiopathic rapid eye movement sleep behavior disorder. *Mov. Disord.* 33 88–98. 10.1002/mds.27105 28843021 PMC5811909

[B60] Heneka M. Carson M. El Khoury J. Landreth G. Brosseron F. Feinstein D. et al. (2015). Neuroinflammation in Alzheimer’s disease. *Lancet Neurol.* 14 388–405. 10.1016/S1474-4422(15)70016-5 25792098 PMC5909703

[B61] Hill-Burns E. Debelius J. Morton J. Wissemann W. Lewis M. Wallen Z. et al. (2017). Parkinson’s disease and Parkinson’s disease medications have distinct signatures of the gut microbiome. *Mov. Disord.* 32 739–749. 10.1002/mds.26942 28195358 PMC5469442

[B62] Hindle J. (2010). Ageing, neurodegeneration and Parkinson’s disease. *Age Ageing* 39 156–161. 10.1093/ageing/afp223 20051606

[B63] Hodgkinson K. El Abbar F. Dobranowski P. Manoogian J. Butcher J. Figeys D. et al. (2023). Butyrate’s role in human health and the current progress towards its clinical application to treat gastrointestinal disease. *Clin. Nutr.* 42 61–75. 10.1016/j.clnu.2022.10.024 36502573

[B64] Holden S. Finseth T. Sillau S. Berman B. (2018). Progression of MDS-UPDRS scores over five years in de novo Parkinson disease from the Parkinson’s progression markers initiative cohort. *Mov. Disord. Clin. Pract.* 5 47–53. 10.1002/mdc3.12553 29662921 PMC5898442

[B65] Hopfner F. Künstner A. Müller S. Künzel S. Zeuner K. Margraf N. et al. (2017). Gut microbiota in Parkinson disease in a northern German cohort. *Brain Res.* 1667 41–45. 10.1016/j.brainres.2017.04.019 28506555

[B66] Hou L. Tian H. Wang L. Ferris Z. Wang J. Cai M. et al. (2022). Identification and biosynthesis of pro-inflammatory sulfonolipids from an opportunistic pathogen Chryseobacterium gleum. *ACS Chem. Biol.* 17 1197–1206. 10.1021/acschembio.2c00141 35476918 PMC9275813

[B67] Huang B. Chau S. Liu Y. Chan J. Wang J. Ma S. et al. (2023). Gut microbiome dysbiosis across early Parkinson’s disease, REM sleep behavior disorder and their first-degree relatives. *Nat. Commun.* 14:2501. 10.1038/s41467-023-38248-4 37130861 PMC10154387

[B68] Huang C.-H. Huang C.-T. Tsai H.-Y. Liao Y.-C. Lin C.-M. Chen P.-C. et al. (2024). Distinct protective effects of a novel Akkermansia sp. BCRC 18949 against DSS-Induced colitis in mice. *J. Funct. Foods* 115:106110. 10.1016/j.jff.2024.106110

[B69] Hung Y. Lyu W. Tsai M. Liu C. Lai L. Tsai M. et al. (2022). To compare the performance of prokaryotic taxonomy classifiers using curated 16S full-length rRNA sequences. *Comput. Biol. Med.* 145:105416. 10.1016/j.compbiomed.2022.105416 35313206

[B70] Ilie O. Vãcãrean-Trandafir I. Amãrandi R. Nita I. Dobrin P. Doroftei M. et al. (2025). Exploring gut microbiota alterations in Parkinson’s disease: Insights from a 16S amplicon sequencing Eastern European pilot study. *Front. Neurosci.* 19:1654995. 10.3389/fnins.2025.1654995 41245833 PMC12615422

[B71] Jacob A. Rout S. Dash R. Venugopal G. Punde A. John J. et al. (2025). Gut microbiome differences in Parkinson’s disease patients in Central Kerala population. *Neurodegener. Dis. Manag*. 10.1080/17582024.2025.2574204 Online ahead of print. 41117133 PMC13192096

[B72] Janda J. Abbott S. (2007). 16S rRNA gene sequencing for bacterial identification in the diagnostic laboratory: Pluses, perils, and pitfalls. *J. Clin. Microbiol.* 45 2761–2764. 10.1128/JCM.01228-07 17626177 PMC2045242

[B73] Ji L. Huang T. Mao L. Xu Y. Chen W. Wang W. et al. (2023). The gut microbiota metabolite butyrate mitigates MPTP/MPP+ -induced Parkinson’s disease by inhibiting the JAK2/STAT3 signaling pathway. *Kaohsiung J. Med. Sci.* 39 1002–1010. 10.1002/kjm2.12745 37807941 PMC11895885

[B74] Jin M. Li J. Liu F. Lyu N. Wang K. Wang L. et al. (2019). Analysis of the gut microflora in patients With Parkinson’s disease. *Front. Neurosci.* 13:1184. 10.3389/fnins.2019.01184 31824239 PMC6883725

[B75] Jo S. Kang W. Hwang Y. Lee S. Park K. Kim M. et al. (2022). Oral and gut dysbiosis leads to functional alterations in Parkinson’s disease. *NPJ Parkinsons Dis.* 8:87. 10.1038/s41531-022-00351-6 35798742 PMC9262988

[B76] Jobin C. (2014). GPR109a: The missing link between microbiome and good health? *Immunity* 40 8–10. 10.1016/j.immuni.2013.12.009 24439263 PMC4337780

[B77] Kakoty V. Dubey S. K. Yang C. H. Taliyan R. (2021). Neuroprotective effects of trehalose and sodium butyrate on preformed fibrillar form of α-Synuclein-induced rat model of Parkinson’s disease. *ACS Chem. Neurosci.* 12 2643–2660. 10.1021/acschemneuro.1c00144 34197084

[B78] Kamel F. Tanner C. Umbach D. Hoppin J. Alavanja M. Blair A. et al. (2007). Pesticide exposure and self-reported Parkinson’s disease in the agricultural health study. *Am. J. Epidemiol.* 165 364–374. 10.1093/aje/kwk024 17116648

[B79] Karunaratne T. Okereke C. Seamon M. Purohit S. Wakade C. Sharma A. (2020). Niacin and butyrate: Nutraceuticals targeting dysbiosis and intestinal permeability in Parkinson’s disease. *Nutrients* 13:28. 10.3390/nu13010028 33374784 PMC7824468

[B80] Kelly S. Munoz-Munoz J. van Sinderen D. (2021). Plant glycan metabolism by bifidobacteria. *Front. Microbiol.* 12:609418. 10.3389/fmicb.2021.609418 33613480 PMC7889515

[B81] Kendall G. Underwood C. Parr-Brownlie L. C. (2025). A novel rat model for inflammatory gut-brain interactions in Parkinson’s disease. *Eur. J. Neurosci.* 61:e16667. 10.1111/ejn.16667 39844519 PMC11754928

[B82] Kim B. Choi C. Shin H. Jin S. Bae J. Han M. et al. (2019). Comparison of the gut microbiota of centenarians in longevity villages of South Korea with those of other age groups. *J. Microbiol. Biotechnol.* 29 429–440. 10.4014/jmb.1811.11023 30661321

[B83] Kim S. Kwon S. Kam T. Panicker N. Karuppagounder S. Lee S. et al. (2019). Transneuronal propagation of pathologic α-synuclein from the gut to the brain models Parkinson’s disease. *Neuron* 103 627–641.e7. 10.1016/j.neuron.2019.05.035 31255487 PMC6706297

[B84] Knudsen G. Paulson O. Hertz M. (1991). Kinetic analysis of the human blood-brain barrier transport of lactate and its influence by hypercapnia. *J. Cereb. Blood Flow Metab.* 11 581–586. 10.1038/jcbfm.1991.107 2050746

[B85] Kuczynski J. Lauber C. Walters W. Parfrey L. Clemente J. Gevers D. et al. (2012). Experimental and analytical tools for studying the human microbiome. *Nat. Rev. Genet.* 13 47–58. 10.1038/nrg3129 22179717 PMC5119550

[B86] Langley J. Huddleston D. E. Hu X. P. (2020). “Chapter 20 - Detecting parkinsonian degeneration in lateroventral tier of substantia nigra pars compacta with MRI,” in *Genetics, Neurology, Behavior, and Diet in Parkinson’s Disease*, eds Martin C. R. Preedy V. R. (Cambridge, MA: Academic Press), 313–325.

[B87] Lee H. An J. Kim J. Choi D. Song Y. Lee C. et al. (2022). A Novel bacterium, Butyricimonas virosa, preventing HFD-induced diabetes and metabolic disorders in mice via GLP-1 receptor. *Front. Microbiol.* 13:858192. 10.3389/fmicb.2022.858192 35655996 PMC9152154

[B88] Leeming E. Johnson A. Spector T. Le Roy C. (2019). Effect of diet on the gut microbiota: Rethinking intervention duration. *Nutrients* 11:2862. 10.3390/nu11122862 31766592 PMC6950569

[B89] Lei Y. Tang L. Liu S. Hu S. Wu L. Liu Y. et al. (2021). Parabacteroides produces acetate to alleviate heparanase-exacerbated acute pancreatitis through reducing neutrophil infiltration. *Microbiome* 9:115. 10.1186/s40168-021-01065-2 34016163 PMC8138927

[B90] Leite G. Pimentel M. Barlow G. Mathur R. (2022). The small bowel microbiome changes significantly with age and aspects of the ageing process. *Microb. Cell.* 9 21–23. 10.15698/mic2022.01.768 35083314 PMC8717087

[B91] Li C. Cui L. Yang Y. Miao J. Zhao X. Zhang J. et al. (2019). Gut microbiota differs between Parkinson’s disease patients and healthy controls in Northeast China. *Front. Mol. Neurosci.* 12:171. 10.3389/fnmol.2019.00171 31354427 PMC6637281

[B92] Li F. Wang P. Chen Z. Sui X. Xie X. Zhang J. (2019). Alteration of the fecal microbiota in North-Eastern Han Chinese population with sporadic Parkinson’s disease. *Neurosci. Lett.* 707:134297. 10.1016/j.neulet.2019.134297 31200089

[B93] Li P. Killinger B. Ensink E. Beddows I. Yilmaz A. Lubben N. et al. (2021). Gut microbiota dysbiosis is associated with elevated bile acids in Parkinson’s disease. *Metabolites* 11:29. 10.3390/metabo11010029 33406628 PMC7823437

[B94] Li W. Wu X. Hu X. Wang T. Liang S. Duan Y. et al. (2017). Structural changes of gut microbiota in Parkinson’s disease and its correlation with clinical features. *Sci. China Life Sci.* 60 1223–1233. 10.1007/s11427-016-9001-4 28536926

[B95] Li Z. Hu G. Zhu L. Sun Z. Jiang Y. Gao M. et al. (2021). Study of growth, metabolism, and morphology of Akkermansia muciniphila with an in vitro advanced bionic intestinal reactor. *BMC Microbiol.* 21:61. 10.1186/s12866-021-02111-7 33622254 PMC7901181

[B96] Li Z. Lu G. Luo E. Wu B. Li Z. Guo J. et al. (2022). Oral, nasal, and gut microbiota in Parkinson’s disease. *Neuroscience* 480 65–78. 10.1016/j.neuroscience.2021.10.011 34695538

[B97] Lin A. Zheng W. He Y. Tang W. Wei X. He R. et al. (2018). Gut microbiota in patients with Parkinson’s disease in southern China. *Parkinsonism Relat. Disord.* 53 82–88. 10.1016/j.parkreldis.2018.05.007 29776865

[B98] Lin C. Chen C. Chiang H. Liou J. Chang C. Lu T. et al. (2019). Altered gut microbiota and inflammatory cytokine responses in patients with Parkinson’s disease. *J. Neuroinflammation* 16:129. 10.1186/s12974-019-1528-y 31248424 PMC6598278

[B99] Liu J. Lv X. Ye T. Zhao M. Chen Z. Zhang Y. et al. (2024). Microbiota-microglia crosstalk between Blautia producta and neuroinflammation of Parkinson’s disease: A bench-to-bedside translational approach. *Brain Behav. Immun.* 117 270–282. 10.1016/j.bbi.2024.01.010 38211635

[B100] Liu J. Wang L. Su L. Chen J. Su R. (2025). Exploring the gut microbiota-Parkinson’s disease link: Preliminary insights from metagenomics and Mendelian randomization. *Front. Microbiol.* 16:1654418. 10.3389/fmicb.2025.1654418 41078518 PMC12512240

[B101] Liu S. Chan P. Stoessl A. (2017). The underlying mechanism of prodromal PD: Insights from the parasympathetic nervous system and the olfactory system. *Transl. Neurodegener.* 6:4. 10.1186/s40035-017-0074-8 28239455 PMC5319081

[B102] Liu X. Zou L. Nie C. Qin Y. Tong X. Wang J. et al. (2023). Mendelian randomization analyses reveal causal relationships between the human microbiome and longevity. *Sci. Rep.* 13:5127. 10.1038/s41598-023-31115-8 36991009 PMC10052271

[B103] Lubomski M. Xu X. Holmes A. Muller S. Yang J. Davis R. et al. (2022b). The gut microbiome in Parkinson’s disease: A longitudinal study of the impacts on disease progression and the use of device-assisted therapies. *Front. Aging Neurosci.* 14:875261. 10.3389/fnagi.2022.875261 35656540 PMC9152137

[B104] Lubomski M. Xu X. Holmes A. Muller S. Yang J. Davis R. et al. (2022a). Nutritional intake and gut microbiome composition predict Parkinson’s disease. *Front. Aging Neurosci.* 14:881872. 10.3389/fnagi.2022.881872 35645785 PMC9131011

[B105] Ma J. Yang X. He J. (2024). Comprehensive gut microbiota composition and microbial interactions among the three age groups. *PLoS One* 19:e0305583. 10.1371/journal.pone.0305583 39423213 PMC11488730

[B106] Mackenzie I. R. A. (2001). The pathology of Parkinson’s disease. *Br. Columbia Med. J.* 43 142–147.

[B107] Maffei V. Kim S. Blanchard E. Luo M. Jazwinski S. Taylor C. et al. (2017). Biological aging and the human gut microbiota. *J. Gerontol. A Biol. Sci. Med. Sci.* 72 1474–1482. 10.1093/gerona/glx042 28444190 PMC5861892

[B108] Maini Rekdal V. Bess E. Bisanz J. Turnbaugh P. Balskus E. (2019). Discovery and inhibition of an interspecies gut bacterial pathway for Levodopa metabolism. *Science* 364:eaau6323. 10.1126/science.aau6323 31196984 PMC7745125

[B109] Martínez-Martín P. Rodríguez-Blázquez C. Alvarez M. Arakaki T. Arillo V. C. Chaná P. et al. (2015). Parkinson’s disease severity levels and MDS-unified Parkinson’s disease rating scale. *Parkinsonism Relat. Disord.* 21 50–54. 10.1016/j.parkreldis.2014.10.026 25466406

[B110] Martinez-Medina M. Denizot J. Dreux N. Robin F. Billard E. Bonnet R. et al. (2014). Western diet induces dysbiosis with increased E coli in CEABAC10 mice, alters host barrier function favouring AIEC colonisation. *Gut* 63 116–124. 10.1136/gutjnl-2012-304119 23598352

[B111] Mehanna M. AbuRaya S. Ahmed S. Ashmawy G. Ibrahim A. AbdelKhaliq E. (2023). Study of the gut microbiome in Egyptian patients with Parkinson’s disease. *BMC Microbiol.* 23:196. 10.1186/s12866-023-02933-7 37481569 PMC10362707

[B112] Metcalfe-Roach A. Cirstea M. Yu A. Ramay H. Coker O. Boroomand S. et al. (2024). Metagenomic analysis reveals large-scale disruptions of the gut microbiome in Parkinson’s disease. *Mov. Disord.* 39 1740–1751. 10.1002/mds.29959 39192744

[B113] Mohammadzadeh R. Mahnert A. Shinde T. Kumpitsch C. Weinberger V. Schmidt H. et al. (2025). Age-related dynamics of predominant methanogenic archaea in the human gut microbiome. *BMC Microbiol.* 25:193. 10.1186/s12866-025-03921-9 40181255 PMC11969853

[B114] Morrison D. Preston T. (2016). Formation of short chain fatty acids by the gut microbiota and their impact on human metabolism. *Gut Microbes* 7 189–200. 10.1080/19490976.2015.1134082 26963409 PMC4939913

[B115] Moustafa A. Chakravarthy S. Phillips J. Gupta A. Keri S. Polner B. et al. (2016). Motor symptoms in Parkinson’s disease: A unified framework. *Neurosci. Biobehav. Rev.* 68 727–740. 10.1016/j.neubiorev.2016.07.010 27422450

[B116] Movement Disorder Society Task Force on Rating Scales for Parkinson’s Disease. (2003). The unified Parkinson’s disease rating scale (UPDRS): Status and recommendations. *Mov. Disord.* 18 738–750. 10.1002/mds.10473 12815652

[B117] Nagatsu T. Sawada M. (2005). Inflammatory process in Parkinson’s disease: Role for cytokines. *Curr. Pharm. Des.* 11 999–1016. 10.2174/1381612053381620 15777250

[B118] Ni Y. Tong Q. Xu M. Gu J. Ye H. (2025). Gut microbiota-induced modulation of the central nervous system function in Parkinson’s disease through the gut-brain axis and short-chain fatty acids. *Mol. Neurobiol.* 62 2480–2492. 10.1007/s12035-024-04370-7 39134825

[B119] Ning J. Huang S. Chen S. Zhang Y. Huang Y. Yu J. (2022). Investigating casual associations among gut microbiota, metabolites, and neurodegenerative diseases: A mendelian randomization study. *J. Alzheimers Dis.* 87 211–222. 10.3233/JAD-215411 35275534

[B120] Nishiwaki H. Ito M. Ishida T. Hamaguchi T. Maeda T. Kashihara K. et al. (2020). Meta-analysis of gut dysbiosis in Parkinson’s disease. *Mov. Disord.* 35 1626–1635. 10.1002/mds.28119 32557853

[B121] Nuzum N. Szymlek-Gay E. Loke S. Dawson S. Teo W. Hendy A. et al. (2023). Differences in the gut microbiome across typical ageing and in Parkinson’s disease. *Neuropharmacology* 235:109566. 10.1016/j.neuropharm.2023.109566 37150399

[B122] Oh B. Choi W. Kim J. Ryu S. Yu S. Lee J. et al. (2021). Cell-free supernatant of Odoribacter splanchnicus isolated from human feces exhibits anti-colorectal cancer activity. *Front. Microbiol.* 12:736343. 10.3389/fmicb.2021.736343 34867852 PMC8638082

[B123] Okazaki Y. Katayama T. (2021). The effects of different high-fat (lard, soybean oil, corn oil or olive oil) diets supplemented with fructo-oligosaccharides on colonic alkaline phosphatase activity in rats. *Eur. J. Nutr.* 60 89–99. 10.1007/s00394-020-02219-y 32193633

[B124] Ozato N. Saito S. Yamaguchi T. Katashima M. Tokuda I. Sawada K. et al. (2019). Blautia genus associated with visceral fat accumulation in adults 20-76 years of age. *NPJ Biofilms Microbiomes* 5:28. 10.1038/s41522-019-0101-x 31602309 PMC6778088

[B125] Palacios N. Hannoun A. Flahive J. Ward D. Goostrey K. Deb A. et al. (2021). Effect of levodopa initiation on the gut microbiota in Parkinson’s disease. *Front. Neurol.* 12:574529. 10.3389/fneur.2021.574529 33746867 PMC7970035

[B126] Palacios N. Wilkinson J. Bjornevik K. Schwarzschild M. McIver L. Ascherio A. et al. (2023). Metagenomics of the gut microbiome in Parkinson’s disease: Prodromal changes. *Ann. Neurol.* 94 486–501. 10.1002/ana.26719 37314861 PMC10538421

[B127] Palmas V. Pisanu S. Madau V. Casula E. Deledda A. Cusano R. et al. (2022). Gut microbiota markers and dietary habits associated with extreme longevity in healthy sardinian centenarians. *Nutrients* 14:2436. 10.3390/nu14122436 35745166 PMC9227524

[B128] Pang S. Chen X. Lu Z. Meng L. Huang Y. Yu X. et al. (2023). Longevity of centenarians is reflected by the gut microbiome with youth-associated signatures. *Nat. Aging* 3 436–449. 10.1038/s43587-023-00389-y 37117794

[B129] Papić E. Rački V. Hero M. Zimani A. Čižek Sajko M. Rožmarić G. et al. (2025). Microbial diversity in drug-naïve Parkinson’s disease patients. *PLoS One* 20:e0328761. 10.1371/journal.pone.0328761 40824914 PMC12360607

[B130] Park D. Kang W. Shin I. Chalita M. Oh H. Hyun D. et al. (2024). Difference in gut microbial dysbiotic patterns between body-first and brain-first Parkinson’s disease. *Neurobiol. Dis.* 201:106655. 10.1016/j.nbd.2024.106655 39218360

[B131] Parker B. Wearsch P. Veloo A. Rodriguez-Palacios A. (2020). The genus alistipes: Gut bacteria with emerging implications to inflammation, cancer, and mental health. *Front. Immunol.* 11:906. 10.3389/fimmu.2020.00906 32582143 PMC7296073

[B132] Pavan S. Gorthi S. Prabhu A. Das B. Mutreja A. Vasudevan K. et al. (2023). Dysbiosis of the beneficial gut bacteria in patients with Parkinson’s disease from India. *Ann. Indian Acad. Neurol.* 26 908–916. 10.4103/aian.aian_460_23 38229613 PMC10789430

[B133] Pellegrini C. D’Antongiovanni V. Miraglia F. Rota L. Benvenuti L. Di Salvo C. et al. (2022). Enteric α-synuclein impairs intestinal epithelial barrier through caspase-1-inflammasome signaling in Parkinson’s disease before brain pathology. *NPJ Parkinsons Dis.* 8:9. 10.1038/s41531-021-00263-x 35022395 PMC8755783

[B134] Petrov V. Saltykova I. Zhukova I. Alifirova V. Zhukova N. Dorofeeva Y. et al. (2017). Analysis of gut microbiota in patients with Parkinson’s disease. *Bull. Exp. Biol. Med.* 162 734–737. 10.1007/s10517-017-3700-7 28429209

[B135] Pfaffinger J. Hays K. Seeley J. Ramesh Babu P. Ryznar R. (2025). Gut dysbiosis as a potential driver of Parkinson’s and Alzheimer’s disease pathogenesis. *Front. Neurosci.* 19:1600148. 10.3389/fnins.2025.1600148 40880851 PMC12380846

[B136] Pietrucci D. Cerroni R. Unida V. Farcomeni A. Pierantozzi M. Mercuri N. et al. (2019). Dysbiosis of gut microbiota in a selected population of Parkinson’s patients. *Parkinsonism Relat. Disord.* 65 124–130. 10.1016/j.parkreldis.2019.06.003 31174953

[B137] Postuma R. Berg D. Stern M. Poewe W. Olanow C. Oertel W. et al. (2015). MDS clinical diagnostic criteria for Parkinson’s disease. *Mov. Disord.* 30 1591–1601. 10.1002/mds.26424 26474316

[B138] Qian Y. Yang X. Xu S. Wu C. Song Y. Qin N. et al. (2018). Alteration of the fecal microbiota in Chinese patients with Parkinson’s disease. *Brain Behav. Immun.* 70 194–202. 10.1016/j.bbi.2018.02.016 29501802

[B139] Reeve A. Simcox E. Turnbull D. (2014). Ageing and Parkinson’s disease: Why is advancing age the biggest risk factor? *Ageing Res. Rev.* 14 19–30. 10.1016/j.arr.2014.01.004 24503004 PMC3989046

[B140] Ren T. Gao Y. Qiu Y. Jiang S. Zhang Q. Zhang J. et al. (2020). Gut microbiota altered in mild cognitive impairment compared with normal cognition in sporadic Parkinson’s disease. *Front. Neurol.* 11:137. 10.3389/fneur.2020.00137 32161568 PMC7052381

[B141] Ren W. Yan H. Yu B. Walsh M. Yu J. Zheng P. et al. (2021). Prevotella- rich enterotype may benefit gut health in finishing pigs fed diet with a high amylose-to-amylopectin ratio. *Anim. Nutr.* 7 400–411. 10.1016/j.aninu.2020.08.007 34258428 PMC8245826

[B142] Rietdijk C. Perez-Pardo P. Garssen J. van Wezel R. Kraneveld A. (2017). Exploring Braak’s hypothesis of Parkinson’s disease. *Front. Neurol.* 8:37. 10.3389/fneur.2017.00037 28243222 PMC5304413

[B143] Ring C. Klopfleisch R. Dahlke K. Basic M. Bleich A. Blaut M. (2019). Akkermansia muciniphila strain ATCC BAA-835 does not promote short-term intestinal inflammation in gnotobiotic interleukin-10-deficient mice. *Gut Microbes* 10 188–203. 10.1080/19490976.2018.1511663 30252588 PMC6546315

[B144] Romano S. Savva G. Bedarf J. Charles I. Hildebrand F. Narbad A. (2021). Meta-analysis of the Parkinson’s disease gut microbiome suggests alterations linked to intestinal inflammation. *NPJ Parkinsons Dis.* 7:27. 10.1038/s41531-021-00156-z 33692356 PMC7946946

[B145] Romo-Vaquero M. Fernández-Villalba E. Gil-Martinez A. Cuenca-Bermejo L. Espín J. Herrero M. et al. (2022). Urolithins: Potential biomarkers of gut dysbiosis and disease stage in Parkinson’s patients. *Food Funct.* 13 6306–6316. 10.1039/d2fo00552b 35611932

[B146] Ross G. Abbott R. Petrovitch H. Tanner C. White L. (2012). Pre-motor features of Parkinson’s disease: The Honolulu-Asia aging study experience. *Parkinsonism Relat. Disord.* 18 S199–S202. 10.1016/S1353-8020(11)70062-1 22166434

[B147] Rust C. van den Heuvel L. Bardien S. Carr J. Pretorius E. Seedat S. et al. (2025). Association between the relative abundance of butyrate-producing and mucin-degrading taxa and Parkinson’s disease. *Neuroscience* 576 149–154. 10.1016/j.neuroscience.2025.04.050 40318838

[B148] Sampson T. Debelius J. Thron T. Janssen S. Shastri G. Ilhan Z. et al. (2016). Gut microbiota regulate motor deficits and neuroinflammation in a model of Parkinson’s disease. *Cell* 167 1469–1480.e12. 10.1016/j.cell.2016.11.018 27912057 PMC5718049

[B149] Scheperjans F. Levo R. Bosch B. Lääperi M. Pereira P. Smolander O. et al. (2024). Fecal microbiota transplantation for treatment of Parkinson disease: A randomized clinical trial. *JAMA Neurol.* 81 925–938. 10.1001/jamaneurol.2024.2305 39073834 PMC11287445

[B150] Schmitt M. Soergel K. Wood C. (1976). Absorption of short chain fatty acids from the human jejunum. *Gastroenterology* 70 211–215. 1248680

[B151] Sepp E. Smidt I. Rööp T. Štšepetova J. Kõljalg S. Mikelsaar M. et al. (2022). Comparative analysis of gut microbiota in centenarians and young people: Impact of eating habits and childhood living environment. *Front. Cell. Infect. Microbiol.* 12:851404. 10.3389/fcimb.2022.851404 35372105 PMC8965453

[B152] Shalash A. Ezzeldin S. Hashish S. Salah Y. Dawood N. Moustafa A. et al. (2025). Gut microbial shifts toward inflammation in Parkinson’s disease: Insights from pilot shotgun metagenomics Egyptian cohort. *J. Parkinsons Dis.* 15 1540–1543. 10.1177/1877718X251370156 40961238 PMC13347530

[B153] Shankar V. Gouda M. Moncivaiz J. Gordon A. Reo N. Hussein L. et al. (2017). Differences in gut metabolites and microbial composition and functions between Egyptian and U.S. children are consistent with their diets. *mSystems* 2:e00169-16. 10.1128/mSystems.00169-16 28191503 PMC5296411

[B154] Shen T. Yue Y. He T. Huang C. Qu B. Lv W. et al. (2021). The association between the gut microbiota and Parkinson’s disease, a meta-analysis. *Front. Aging Neurosci.* 13:636545. 10.3389/fnagi.2021.636545 33643026 PMC7907649

[B155] Stagaman K. Kmiecik M. Wetzel M. Aslibekyan S. Sonmez T. Fontanillas P. et al. (2024). Oral and gut microbiome profiles in people with early idiopathic Parkinson’s disease. *Commun. Med.* 4:209. 10.1038/s43856-024-00630-8 39443634 PMC11499922

[B156] Sun L. Li Z. Hu C. Ding J. Zhou Q. Pang G. et al. (2023). Age-dependent changes in the gut microbiota and serum metabolome correlate with renal function and human aging. *Aging Cell.* 22:e14028. 10.1111/acel.14028 38015106 PMC10726799

[B157] Tan A. Chong C. Lim S. Yap I. Teh C. Loke M. et al. (2021). Gut microbial ecosystem in Parkinson disease: New clinicobiological insights from multi-omics. *Ann. Neurol.* 89 546–559. 10.1002/ana.25982 33274480

[B158] Tan A. Lim S. Lang A. (2022). The microbiome-gut-brain axis in Parkinson disease - from basic research to the clinic. *Nat. Rev. Neurol.* 18 476–495. 10.1038/s41582-022-00681-2 35750883

[B159] Tizabi Y. Getachew B. Aschner M. (2023). Butyrate protects and synergizes with nicotine against iron- and manganese-induced toxicities in cell culture. *Neurotox Res.* 42:3. 10.1007/s12640-023-00682-z 38095760

[B160] Toh T. Chong C. Lim S. Bowman J. Cirstea M. Lin C. et al. (2022). Gut microbiome in Parkinson’s disease: New insights from meta-analysis. *Parkinsonism Relat. Disord.* 94 1–9. 10.1016/j.parkreldis.2021.11.017 34844021

[B161] Tuikhar N. Keisam S. Labala R. Imrat, Ramakrishnan P. Arunkumar M. C. et al. (2019). Comparative analysis of the gut microbiota in centenarians and young adults shows a common signature across genotypically non-related populations. *Mech. Ageing Dev.* 179 23–35. 10.1016/j.mad.2019.02.001 30738080

[B162] Tursi A. Procaccianti G. De Bastiani R. Turroni S. D’Amico F. Allegretta L. et al. (2025). Micro-encapsulated and colonic-release sodium butyrate modulates gut microbiota and improves abdominal pain in patients with symptomatic uncomplicated diverticular disease. *Front. Med.* 12:1487892. 10.3389/fmed.2025.1487892 40078388 PMC11897004

[B163] Van Den Berge N. Ulusoy A. (2022). Animal models of brain-first and body-first Parkinson’s disease. *Neurobiol. Dis.* 163:105599. 10.1016/j.nbd.2021.105599 34952161

[B164] Vernero M. De Blasio F. Ribaldone D. Bugianesi E. Pellicano R. Saracco G. et al. (2020). The usefulness of microencapsulated sodium butyrate add-on therapy in maintaining remission in patients with ulcerative colitis: A prospective observational study. *J. Clin. Med.* 9:3941. 10.3390/jcm9123941 33291846 PMC7762036

[B165] Villette R. Novikova P. Laczny C. Mollenhauer B. May P. Wilmes P. (2025a). Human gut microbiome gene co-expression network reveals a loss in taxonomic and functional diversity in Parkinson’s disease. *NPJ Biofilms Microbiomes* 11:142. 10.1038/s41522-025-00780-0 40707492 PMC12289924

[B166] Villette R. Ortís Sunyer J. Novikova P. Aho V. Petrov V. Hickl O. et al. (2025b). Integrated multi-omics highlights alterations of gut microbiome functions in prodromal and idiopathic Parkinson’s disease. *Microbiome* 13:200. 10.1186/s40168-025-02227-2 41053825 PMC12502400

[B167] Vincent A. Wang X. Parsons S. Khan W. Huizinga J. (2018). Abnormal absorptive colonic motor activity in germ-free mice is rectified by butyrate, an effect possibly mediated by mucosal serotonin. *Am. J. Physiol. Gastrointest. Liver Physiol.* 315 G896–G907. 10.1152/ajpgi.00237.2017 30095295

[B168] Vitetta L. Llewellyn H. Oldfield D. (2019). Gut dysbiosis and the intestinal microbiome: Streptococcus thermophilus a key probiotic for reducing uremia. *Microorganisms* 7:228. 10.3390/microorganisms7080228 31370220 PMC6723445

[B169] Walker A. Pfitzner B. Harir M. Schaubeck M. Calasan J. Heinzmann S. et al. (2017). Sulfonolipids as novel metabolite markers of alistipes and odoribacter affected by high-fat diets. *Sci. Rep.* 7:11047. 10.1038/s41598-017-10369-z 28887494 PMC5591296

[B170] Wallen Z. Appah M. Dean M. Sesler C. Factor S. Molho E. et al. (2020). Characterizing dysbiosis of gut microbiome in PD: Evidence for overabundance of opportunistic pathogens. *NPJ Parkinsons Dis.* 6:11. 10.1038/s41531-020-0112-6 32566740 PMC7293233

[B171] Wallen Z. Demirkan A. Twa G. Cohen G. Dean M. Standaert D. et al. (2022). Metagenomics of Parkinson’s disease implicates the gut microbiome in multiple disease mechanisms. *Nat. Commun.* 13:6958. 10.1038/s41467-022-34667-x 36376318 PMC9663292

[B172] Wang C. Tang Y. Yang T. Wang Y. Niu Z. Zhang K. et al. (2025). Causal relationship between intestinal microbiota, inflammatory cytokines, peripheral immune cells, plasma metabolome and Parkinson’s disease: A mediation mendelian randomization study. *Eur. J. Neurosci.* 61:e16665. 10.1111/ejn.16665 39831637

[B173] Wang J. Qie J. Zhu D. Zhang X. Zhang Q. Xu Y. et al. (2022). The landscape in the gut microbiome of long-lived families reveals new insights on longevity and aging - relevant neural and immune function. *Gut Microbes* 14 2107288. 10.1080/19490976.2022.2107288 35939616 PMC9361766

[B174] Wang K. Wu W. Wang Q. Yang L. Bian X. Jiang X. et al. (2022). The negative effect of Akkermansia muciniphila-mediated post-antibiotic reconstitution of the gut microbiota on the development of colitis-associated colorectal cancer in mice. *Front. Microbiol.* 13:932047. 10.3389/fmicb.2022.932047 36312913 PMC9614165

[B175] Wang N. Li R. Lin H. Fu C. Wang X. Zhang Y. et al. (2019). Enriched taxa were found among the gut microbiota of centenarians in East China. *PLoS One* 14:e0222763. 10.1371/journal.pone.0222763 31639130 PMC6804974

[B176] Wang Q. Luo Y. Ray Chaudhuri K. Reynolds R. Tan E. Pettersson S. (2021). The role of gut dysbiosis in Parkinson’s disease: Mechanistic insights and therapeutic options. *Brain* 144 2571–2593. 10.1093/brain/awab156 33856024

[B177] Weis S. Schwiertz A. Unger M. Becker A. Faßbender K. Ratering S. et al. (2019). Effect of Parkinson’s disease and related medications on the composition of the fecal bacterial microbiota. *NPJ Parkinsons Dis.* 5:28. 10.1038/s41531-019-0100-x 31815177 PMC6884491

[B178] Wu J. Ren W. Chen L. Lou Y. Liu C. Huang Y. et al. (2022). Age-Related Changes in the Composition of Intestinal Microbiota in Elderly Chinese Individuals. *Gerontology* 68 976–988. 10.1159/000520054 35100593

[B179] Wu L. Xie X. Li Y. Liang T. Zhong H. Yang L. et al. (2022). Gut microbiota as an antioxidant system in centenarians associated with high antioxidant activities of gut-resident Lactobacillus. *NPJ Biofilms Microbiomes* 8:102. 10.1038/s41522-022-00366-0 36564415 PMC9789086

[B180] Wu L. Zeng T. Zinellu A. Rubino S. Kelvin D. Carru C. (2019). A cross-sectional study of compositional and functional profiles of gut microbiota in sardinian centenarians. *mSystems* 4:e00325-19. 10.1128/mSystems.00325-19 31289141 PMC6616150

[B181] Xie A. Ensink E. Li P. Gordevièius J. Marshall L. George S. et al. (2022). Bacterial butyrate in Parkinson’s disease is linked to epigenetic changes and depressive symptoms. *Mov. Disord.* 37 1644–1653. 10.1002/mds.29128 35723531 PMC9545646

[B182] Xu K. Wang G. Gong J. Yang X. Cheng Y. Li D. et al. (2025). Akkermansia muciniphila protects against dopamine neurotoxicity by modulating butyrate to inhibit microglia-mediated neuroinflammation. *Int. Immunopharmacol.* 152:114374. 10.1016/j.intimp.2025.114374 40056512

[B183] Xu R. Miao W. Xu J. Xu W. Liu M. Ding S. et al. (2022). Neuroprotective effects of sodium butyrate and Monomethyl fumarate treatment through GPR109A modulation and intestinal barrier restoration on PD mice. *Nutrients* 14:4163. 10.3390/nu14194163 36235813 PMC9571500

[B184] Xu Y. Wen L. Tang Y. Zhao Z. Xu M. Wang T. et al. (2024). Sodium butyrate activates the KATP channels to regulate the mechanism of Parkinson’s disease microglia model inflammation. *Immun. Inflamm. Dis.* 12:e1194. 10.1002/iid3.1194 38501544 PMC10949401

[B185] Yamashita M. Okubo H. Kobuke K. Ohno H. Oki K. Yoneda M. et al. (2019). Alteration of gut microbiota by a Westernized lifestyle and its correlation with insulin resistance in non-diabetic Japanese men. *J. Diabetes Investig.* 10 1463–1470. 10.1111/jdi.13048 30901505 PMC6825921

[B186] Yan J. Feng X. Zhou X. Zhao M. Xiao H. Li R. et al. (2022). Identification of gut metabolites associated with Parkinson’s disease using bioinformatic analyses. *Front. Aging Neurosci.* 14:927625. 10.3389/fnagi.2022.927625 35959296 PMC9360421

[B187] Yan Z. Zhao G. (2024). The associations among gut microbiota, branched chain amino acids, and Parkinson’s disease: Mendelian randomization study. *J. Parkinsons Dis.* 14 1129–1138. 10.3233/JPD-240244 39177611 PMC11380289

[B188] Yang L. Wang Y. Zhang Y. Li W. Jiang S. Qian D. et al. (2022). Gut microbiota: A new avenue to reveal pathological mechanisms of constipation. *Appl. Microbiol. Biotechnol.* 106 6899–6913. 10.1007/s00253-022-12197-2 36190540

[B189] Yoon H. Kang W. Jo S. Hwang Y. Lee J. Chung S. et al. (2024). Dietary quality and the gut microbiome in early-stage Parkinson’s disease patients. *Nutr. Neurosci.* 27 761–769. 10.1080/1028415X.2023.2253025 37711026

[B190] Yuan X. Chen Y. Liu Z. (2024). Relationship among Parkinson’s disease, constipation, microbes, and microbiological therapy. *World J. Gastroenterol.* 30 225–237. 10.3748/wjg.v30.i3.225 38314132 PMC10835526

[B191] Zhang F. Yue L. Fang X. Wang G. Li C. Sun X. et al. (2020). Altered gut microbiota in Parkinson’s disease patients/healthy spouses and its association with clinical features. *Parkinsonism Relat. Disord.* 81 84–88. 10.1016/j.parkreldis.2020.10.034 33099131

[B192] Zhang K. Paul K. Jacobs J. Chou H. Duarte Folle A. Del Rosario I. et al. (2022). Parkinson’s disease and the gut microbiome in rural california. *J. Parkinsons Dis.* 12 2441–2452. 10.3233/JPD-223500 36442206 PMC9890728

[B193] Zhang L. Yuan W. Ye M. Yin L. Wang S. (2023). Changes in the intestinal microbiota of patients with Parkinson’s disease and their clinical significance. *Int. J. Clin. Pharmacol. Ther.* 61 48–58. 10.5414/CP204285 36420886

[B194] Zhang Y. Mo C. He X. Xiao Q. Yang X. (2025). Gut microbial community of patients with Parkinson’s disease analyzed using metagenome-assembled genomes. *Neural Regen. Res.* 10.4103/NRR.NRR-D-25-00420 Online ahead of print. 41017724 PMC13460800

[B195] Zhang Y. Xu S. Qian Y. He X. Mo C. Yang X. et al. (2022). Sodium butyrate attenuates rotenone-induced toxicity by activation of autophagy through epigenetically regulating PGC-1α expression in PC12 cells. *Brain Res.* 1776 147749. 10.1016/j.brainres.2021.147749 34896331

[B196] Zhang Y. Xu S. Qian Y. Mo C. Ai P. Yang X. et al. (2023). Sodium butyrate ameliorates gut dysfunction and motor deficits in a mouse model of Parkinson’s disease by regulating gut microbiota. *Front. Aging Neurosci.* 15:1099018. 10.3389/fnagi.2023.1099018 36761177 PMC9905700

[B197] Zhao Y. Wee H. Chan Y. Seah S. Au W. Lau P. et al. (2010). Progression of Parkinson’s disease as evaluated by Hoehn and Yahr stage transition times. *Mov. Disord.* 25 710–716. 10.1002/mds.22875 20213822

[B198] Zhao Z. Chen J. Zhao D. Chen B. Wang Q. Li Y. et al. (2024). Microbial biomarker discovery in Parkinson’s disease through a network-based approach. *NPJ Parkinsons Dis.* 10:203. 10.1038/s41531-024-00802-2 39461950 PMC11513973

[B199] Zhong Z. Ye M. Yan F. (2023). A review of studies on gut microbiota and levodopa metabolism. *Front. Neurol.* 14:1046910. 10.3389/fneur.2023.1046910 37332996 PMC10272754

[B200] Zhu M. Liu X. Ye Y. Yan X. Cheng Y. Zhao L. et al. (2022). Gut microbiota: A novel therapeutic target for Parkinson’s disease. *Front. Immunol.* 13:937555. 10.3389/fimmu.2022.937555 35812394 PMC9263276

